# Shear relaxation governs fusion dynamics of biomolecular condensates

**DOI:** 10.1038/s41467-021-26274-z

**Published:** 2021-10-13

**Authors:** Archishman Ghosh, Divya Kota, Huan-Xiang Zhou

**Affiliations:** 1 Department of Chemistry, University of Illinois at Chicago, Chicago, IL 60607 USA; 2 Department of Physics, University of Illinois at Chicago, Chicago, IL 60607 USA

**Keywords:** Biopolymers in vivo, Intrinsically disordered proteins

## Abstract

Phase-separated biomolecular condensates must respond agilely to biochemical and environmental cues in performing their wide-ranging cellular functions, but our understanding of condensate dynamics is lagging. Ample evidence now indicates biomolecular condensates as viscoelastic fluids, where shear stress relaxes at a finite rate, not instantaneously as in viscous liquids. Yet the fusion dynamics of condensate droplets has only been modeled based on viscous liquids, with fusion time given by the viscocapillary ratio (viscosity over interfacial tension). Here we used optically trapped polystyrene beads to measure the viscous and elastic moduli and the interfacial tensions of four types of droplets. Our results challenge the viscocapillary model, and reveal that the relaxation of shear stress governs fusion dynamics. These findings likely have implications for other dynamic processes such as multiphase organization, assembly and disassembly, and aging.

## Abstract (web summary)

Phase-separated biomolecular condensates are implicated in a myriad of biological processes. Here the authors apply optical tweezers to characterize the viscoelasticity and interfacial tension of a range of condensates, finding that condensates can deviate from simple fluids in opposite directions; and identify shear relaxation as a governing measure of condensate dynamics.

## Introduction

Phase-separated biomolecular condensates mediate cellular functions ranging from stress response to chromatin organization^1–3^. Condensates, including membraneless organelles such as stress granules^1,2^, P granules^4,5^, and nucleoli^6^, typically appear as micro-sized droplets and span material states from liquid-like to solid-like, or become solid-like over time (a process known as maturation or aging^7–11^). In stress granules and many other cases, a dynamic, liquid state allows for rapid assembly, disassembly, or clearance in response to biochemical or environmental cues and for easy exchange of ligands or macromolecular components with the surrounding bulk phase^1,2,4,12–14^. Condensate solidification has been implicated in neurodegeneration and other pathologies^1,7,9,14,15^. Thus an ATP-dependent process actively maintains the dynamics of nucleoli^12^, and RNA and chaperones prevent condensates from solidification^1,16,17^. In other cases, the extent of liquidity is tuned either temporally or spatially for appropriate condensate assembly or function^5,8,18,19^. Despite the crucial importance of condensate dynamics, its quantification and relation to molecular properties are still elusive.

**Fig. 1:**
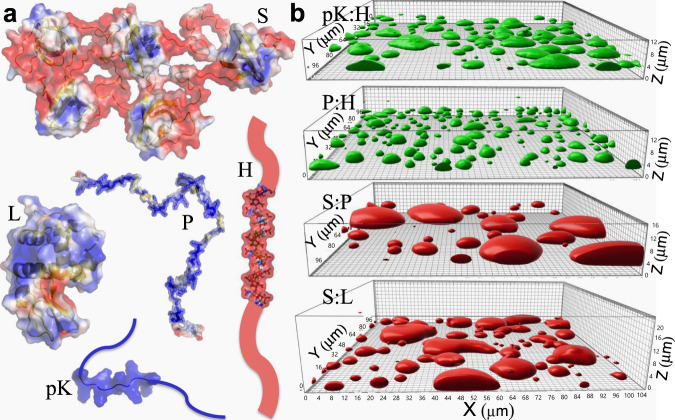
Macromolecular components and the resulting four types of droplets. **a** Molecular structures of pentameric constructs of SH3 domains (S) and proline-rich motifs (P), lysozyme (L), heparin (H), and polylysine (pK), rendered by electrostatic surfaces (blue and red: positive and negative electrostatic potentials, respectively). L is fully folded, P, H, and pK are fully disordered, and S contains both folded domains and disordered linkers. **b** pK:H, P:H, S:P, and S:L droplets settled on a coverslip, visualized by the fluorescence of either FITC-labeled H (green) or Alexa 594-labeled S (red), reproduced from ref. ^26^. The growing tallness of droplets in the series gives a crude indication of a modest increase in interfacial tension from pK:H to S:L.

Liquid droplets have a tendency to fuse and relax into a spherical shape, and the fusion speed has been used as an indicator of condensate dynamics^4,6,7,9,12,18,20–27^. All fusion data have been analyzed by modeling condensates as purely viscous (i.e., Newtonian) liquids, where fusion, driven by interfacial tension (capillarity; $\gamma$) but retarded by viscosity ($\eta$), occurs on the viscocapillary timescale ${\tau }_{{{{{{\rm{vc}}}}}}}=\eta R/\gamma$, where $R$ denotes droplet radius. This viscocapillary model based on Newtonian fluids has not been tested by measuring fusion speed, viscosity, and interfacial tension on the same system. Recent theoretical calculations have cast doubt on its validity if condensates are viscoelastic, i.e., partly liquid and partly solid^28^. A crucial difference between viscous liquids and elastic solids lies in the shear relaxation modulus, $G(t)$, which measures how shear stress relaxes upon the introduction of a unit-step strain at time $t$ = 0 (see Supplementary Text). In many ways $G(t)$ is similar to the memory kernel of a generalized Langevin particle. In viscous liquids, shear relaxation is instantaneous and $G(t)$ is a delta function of time, $\eta \delta (t)$. In elastic solids, shear stress never relaxes and hence $G(t)$ is a constant. In viscoelastic fluids, shear relaxation occurs at a finite rate, as exemplified by the Maxwell fluid, with $G(t)$ given by an exponentially decaying function of time (Supplementary Fig. 1). The Fourier transform of $G(t)$ defines the complex shear modulus ${G}^{\ast }\left(\omega \right)$, whose real and imaginary parts are the elastic and viscous moduli, respectively. Here $\omega$ denotes the angular frequency. ${G}^{\ast }\left(\omega \right)$ data have been reported for some condensates, indicating that they are indeed viscoelastic on the ms to s timescales^10,29–32^. To assess the viscocapillary model and to uncover other determinants of the fusion speed, here we used optical tweezers (OT) to measure the complex shear moduli and the interfacial tensions of diverse condensates. The fusion speeds of these condensates, spanning two orders of magnitude, were reported previously^26^. They were chosen to exemplify the varied structural order of biomolecules of in vivo condensates, encompassing fully folded species, fully disordered species, and species comprising folded domains and disordered regions. The degree of structural order (or compactness) affects how the molecules associate with each (e.g., inter-domain interface vs. interchain bridge) in condensates^26^. The macromolecules in this study (Fig. 1a) include the single-domain protein lysozyme (L), pentameric constructs of SH3 domains (S) and SH3-targeting proline-rich motifs (P), and two polymers: polylysine (pK) and heparin (H). L, P, and pK carry significant net positive charges whereas S and H carry significant negative charges. The four oppositely charged binary mixtures, pK:H, p:H, S:P, and S:L, form droplets, which fall under gravity, fuse, and settle on a coverslip with tallness sustained by interfacial tension (Fig. 1b).

## Results

**Fig. 2:**
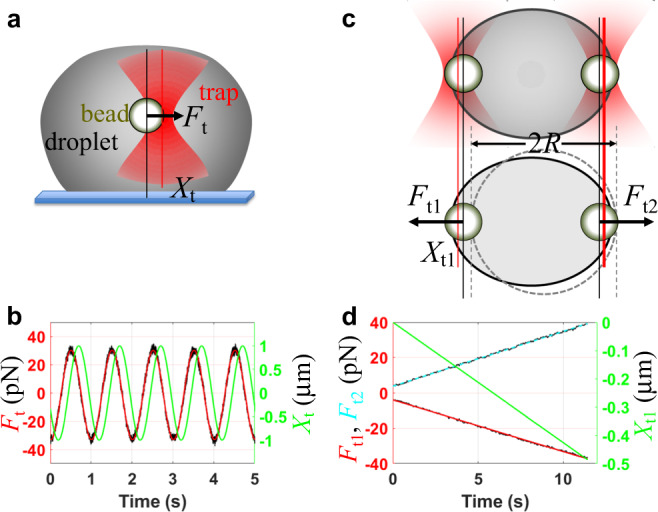
Design of experiments for measuring viscous and elastic moduli and interfacial tension. **a** Oscillatory micro-rheology inside a settled droplet. **b** Time traces of the trap position (*X*_t_; green) and trapping force (*F*_t_; black) on a bead inside a pK:H droplet. A fit of the force trace to a cosine function of time is shown in red. The oscillation frequency (*ω*/2*π*) was 1 Hz. **c** A droplet suspended by two trapped beads at the opposite poles (top: side view; bottom, top view). Trap 2 was fixed in place while trap 1 was pulled toward the left. **d** Traces of the trap 1 position (*X*_t1_; green) and traps 1 and 2 forces (*F*_t1_ and *F*_t2_; black) measured on a pK:H droplet. The force traces were smoothed by moving average over a 64-ms window; linear fits are overlaid. Trap 1 was pulled at a constant speed of ~0.05 μm/s.

**Fig. 3:**
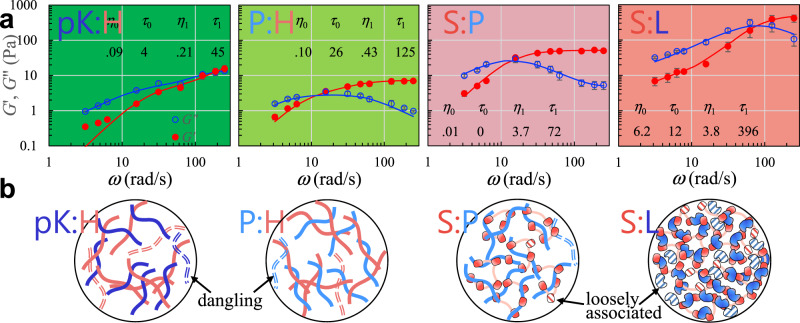
Complex shear moduli and macromolecular networks of four types of droplets. **a** Elastic (*G*′; red filled circles) and viscous (*G*″; blue open circles) moduli of pK:H, P:H, S:P, and S:L droplets. Data were presented as mean ± standard error of the mean (*N* = 3 or 4 replicate measurements). The trap stiffnesses were 310 pN/μm for pK:H, P:H, and S:P droplets and 1850 pN/μm for S:L droplets. Fits of the data to the Burgers model (Eq. 2a, b) are shown as red and blue curves. The fitting parameters ($\eta$ in Pa s and $\tau$ in ms) are listed in the figures. **b** Illustration of macromolecular networks inside droplets. Dangling polymer chains are shown as a dash; loosely associated domains are shown as hatched fill. Successive increases in macromolecular packing density from pK:H and P:H to S:P and to S:L are supported by thioflavin T fluorescence intensity observed on a confocal microscope^26^.

We probed the viscoelasticity inside the settled droplets by oscillating an optically trapped polystyrene bead^33,34^ (Fig. 2a, b and Supplementary Fig. 2a; see Supplementary Information (including Supplementary Fig. 3) for details and Supplementary Movie 1 for illustration). The resulting shear relaxation moduli fit well to a combination of two Maxwell components (Fig. 3a),

1 $$G\left(t\right)=\frac{{\eta }_{0}}{{\tau }_{0}}{e}^{-t/{\tau }_{0}}+\frac{{\eta }_{1}}{{\tau }_{1}}{e}^{-t/{\tau }_{1}}\;{{{{{\rm{for}}}}}}\; t\, > \, 0$$

which is known as the Burgers model for linear viscoelasticity. ${\tau }_{0}$ and ${\tau }_{1}$ are relaxation times, and the corresponding amplitudes, ${\eta }_{0}$ and ${\eta }_{1}$, are viscosities. The resulting elastic and viscous moduli are

2a $${G}^{{\prime} }\left(\omega \right)=\frac{{\omega }^{2}{\tau }_{0}{\eta }_{0}}{1+{(\omega {\tau }_{0})}^{2}}+\frac{{\omega }^{2}{\tau }_{1}{\eta }_{1}}{1+{(\omega {\tau }_{1})}^{2}}$$

2b $$G{{{{{\rm{\hbox{''}}}}}}}\left(\omega \right)=\frac{\omega {\eta }_{0}}{1+{(\omega {\tau }_{0})}^{2}}+\frac{\omega {\eta }_{1}}{1+{(\omega {\tau }_{1})}^{2}}$$

According to transient network models for associative polymers, the shorter time constant, ${\tau }_{0}$, is dictated by the conformational dynamics of the macromolecular components, whereas the longer time constant, ${\tau }_{1}$, is dictated by the kinetics of macromolecular transient association and dissociation^35,36^. The amplitude ratio, ${\eta }_{0}/{\eta }_{1}$, is affected by the population ratio of dangling or loosely associated macromolecules to tightly associated ones (Fig. 3b).

In S:P droplets, ${\tau }_{0}$ is 0, which means that the first component of the shear relaxation modulus is actually Newtonian. In the other three types of condensates, ${\tau }_{0}$ is 5- to 35-fold shorter than the corresponding ${\tau }_{1}$. Most condensate dynamic processes of interest (e.g., droplet fusion) occur on timescales longer than ${\tau }_{0}$; then reconfiguration of macromolecular networks inside condensates, with a time constant ${\tau }_{1}$, is the main mode of shear relaxation. Although the inverse of the crossover angular frequency ${\omega }_{{{{{{\rm{x}}}}}}}$ defined by ${G}^{{\prime} }\left({\omega }_{{{{{{\rm{x}}}}}}}\right)=G\mbox{''}\left({\omega }_{{{{{{\rm{x}}}}}}}\right)$ has commonly been identified as the network reconfiguration time (e.g., ref. ^36^), ${\omega }_{{{{{{\rm{x}}}}}}}$ for the Burgers model actually depends on ${\eta }_{0}/{\eta }_{1}$ (Supplementary Fig. 4). $1/{\omega }_{{{{{{\rm{x}}}}}}}$ is close to ${\tau }_{1}$ only when ${\eta }_{0}/{\eta }_{1}$ is <0.26. This condition is not satisfied in two (pK:H and S:L) of the four types of condensates studied, where $1/{\omega }_{{{{{{\rm{x}}}}}}}$ is closer to ${\tau }_{0}$ instead of ${\tau }_{1}$. S:L is the only case where ${\eta }_{0}/{\eta }_{1}$ is >1, likely due to a high level of loosely associated L molecules (Fig. 3b). In all the four types of condensates, the network reconfiguration time ${\tau }_{1}$ is tens to hundreds ms.

**Fig. 4:**
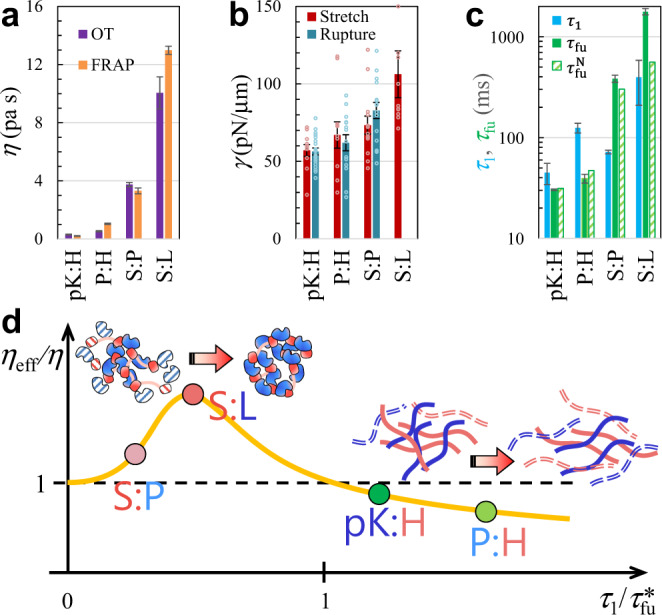
Deviations of condensates from Newtonian fluids. **a** Comparison of zero-shear viscosities from the present OT measurements and deduced from FRAP time constants in our previous study^26^. Results are presented as fitted value ± standard error of the fit. OT results were determined by fitting the data in Fig. 3a to the Burgers model (Eq. 2a, b). FRAP results were converted from FRAP time constants (Eq. S15 in Supplementary Text); the latter in turn were determined by fitting FRAP time traces (average of triplicate measurements) to an exponential function (Eq. S13 in Supplementary Text). For S:L droplets, the L concentration was 300 μM in the OT measurements but 2000 μM in FRAP; a somewhat higher $\eta$ is thus expected in the FRAP experiment. For pK:H droplets, the equimolar component concentration was 100 μM in the OT measurements but 50 μM in FRAP. **b** Interfacial tensions from OT measurements, by either stretching (*N* = 10 to 13) or rupturing (*N* = 14 to 20) droplets. Dot plots display raw data from replicate measurements; bar graphs display mean ± standard error of the mean. The trap stiffnesses were 450 to 750 pN/μm in the stretching experiments and around 1600 pN/μm in the rupture experiments. Quantification of interfacial tension from rupture force involved an arbitrary scaling constant *f* (see Eq. 43 in Supplementary Information). For S:L droplets, the rupture force exceeded the trapping power of the instrument and hence only a lower bound of 100 pN/μm could be placed for $\gamma$. **c** Comparison of shear relaxation time (${\tau }_{1}$; this study), measured fusion time (${\tau }_{{{{{{\rm{fu}}}}}}}$; data were determined here for pK:H and from ref. ^26^ for the other three types of droplets), and fusion time (${\tau }_{{{{{{\rm{fu}}}}}}}^{{{{{{\rm{N}}}}}}}$) predicted by the viscocapillary model. Results for ${\tau }_{1}$ were determined by fitting the data in Fig. 3a to the Burgers model (Eq. 2a, b), and are presented as fitted value ± standard error of the fit. Raw ${\tau }_{{{{{{\rm{fu}}}}}}}$ data (*N* = 4 to 17 droplets with various sizes) were first fit to a linear function of droplet radius (Eq. 3); ${\tau }_{{{{{{\rm{fu}}}}}}}$ results in the bar graph represent fitted slope ± standard error of the fit, both multiplied by a fixed $R$ = 3 μm. **d** Shear thickening at ${\tau }_{1}/{\tau }_{{{{{{\rm{fu}}}}}}}^{\ast }$ < 1 and shear thinning at ${\tau }_{1}/{\tau }_{{{{{{\rm{fu}}}}}}}^{\ast }$ > 1. The solid curve displays characteristic behaviors of associative polymers. The illustration at the top shows a shear-induced strengthening of macromolecular networks; the illustration on the right shows the opposite effect.

In dynamic processes that are much slower than macromolecular network reconfiguration, shear relaxation is fast by comparison and condensates then behave entirely as Newtonian fluids, with viscosity $\eta ={\eta }_{0}+{\eta }_{1}$. Fluorescence recovery after photobleaching (FRAP) is such an example, with time constants (${\tau }_{{{{{{\rm{FR}}}}}}}$) ranging from 2.1 ± 0.2 s to 105.1 ± 2.3 s for pK:H, P:H, S:P, and S:L condensates^26^. Other methods (e.g., particle tracking) that work on similar or longer timescales would also only reveal Newtonian behaviors^6,20,21,27,37–39^. In a Newtonian fluid, ${\tau }_{{{{{{\rm{FR}}}}}}}$ is inversely proportional to the diffusion constant of the fluorescently labeled species and proportional to the fluid viscosity. These measured ${\tau }_{{{{{{\rm{FR}}}}}}}$ values do correlate with the zero-shear viscosities ($\eta$) measured here by OT-based oscillatory micro-rheology, which are 0.30 ± 0.03, 0.53 ± 0.04, 3.75 ± 0.14, and 10.1 ± 1.1 Pa s for the four types of condensates. Indeed, the viscosities deduced from ${\tau }_{{{{{{\rm{FR}}}}}}}$ (see Supplementary Text) show quantitative agreement with the OT-measured values (Fig. 4a). The viscosity of S:L condensates is 33 times higher than that of pK:H condensates. In reference to the viscosity of water (${\eta }_{{{{{{\rm{w}}}}}}}$; = 8.9 × 10^−4^ Pa s at 25 °C), the relative viscosities ($\eta /{\eta }_{{{{{{\rm{w}}}}}}}$) of the four types of condensates are 340, 600, 4200, and 11,000.

Two main determinants of viscosity are macromolecular interaction strength and degree of macromolecular structural compactness^26^. Between pK:H and P:H, the slightly lower viscosity of the former can be attributed to a higher salt concentration (i.e., 1.0 M KCl, which enabled pK:H to form liquid droplets; at the 0.15 M KCl concentration in the other three types of condensates, pK:H only formed network-like aggregates). KCl decreases viscosity directly by screening electrostatic attraction between the oppositely charged components, and likely also indirectly because the weakened attraction could reduce macromolecular concentrations in condensates. When the KCl concentration in P:H condensates is increased from 0.15 to 0.3 and 0.4 M, the zero-shear viscosity decreases from 0.53 ± 0.04 to 0.25 ± 0.02 and 0.22 ± 0.01 Pa s, respectively. The components of pK:H and P:H condensates are polymers, which loosely pack but are connected to each other by numerous interchain bridges (Fig. 3b, left two panels). In contrast, the components of S:L condensates are a single folded domain and a pentamer of folded domains; inter-domain association involves an extensive interface and results in close macromolecular packing (Fig. 3b, right most panel). The increase in viscosity going from P:H to S:P to S:L thus likely in part reflects the rise in the structural compactness of the components, leading to higher packing densities. The relative viscosity of a pure L solution at a high concentration of 480 g/L (or 34 mM) is only 153 at 25 °C^40^. The relative viscosity ${\eta }_{0}/{\eta }_{{{{{{\rm{w}}}}}}}$ attributable to loosely associated L in S:L condensates is 7000. The latter much greater value suggests that even those loosely associated L molecules have significant interactions with surrounding macromolecular networks. In the pure L solution, a decrease in temperature to 5 °C effectively strengthened the attraction among L molecules and increased the relative viscosity to 2200^40^.

Let us now compare the network reconfiguration times (${\tau }_{1}$) among the four types of condensates. pK:H condensates have the shortest ${\tau }_{1}$, at 45 ± 11 ms. One reason again is the high concentration of KCl, which weakens interchain electrostatic interactions and thereby interchains bridges more readily break. This conclusion is supported by the decrease in ${\tau }_{1}$ in P:H condensates, from 125 ± 14 to 56 ± 10 ms and 36 ± 3 ms, respectively, when the KCl concentration is deceased from 0.15 to 0.3 and 0.4 M. S:L condensates have the longest ${\tau }_{1}$, at 396 ± 187 ms. As noted previously^26^, breaking the extensive contacts between folded domains takes more energy and hence occurs more slowly than breaking bridges between polymer chains. S:P condensates have a ${\tau }_{1}$ (72 ± 3 ms) that is shorter than the counterparts in both P:H and S:L condensates. Of the two components of S:P condensates, one is a polymer and the other is a pentamer of folded domains. The resulting macromolecular networks are neither as entangled as in P:H condensates nor as densely packed as in S:L condensates, therefore explaining the shorter ${\tau }_{1}$. We note that in condensates formed by quaternary mixtures of S, P, H, and L, the components demix to generate P:H-rich and S:L-rich foci^41^. This phenomenon is probably related to the rapid breakup of S:P networks as indicated by the short shear relaxation time ${\tau }_{1}$.

We measured the interfacial tensions ($\gamma$) of pK:H, P:H, S:P, and S:L droplets by stretching them using two trapped beads positioned at the opposite poles^30^ (Fig. 2c, d and Supplementary Figs. 2b and 5; see Supplementary Information for details and Supplementary Movie 2 for illustration). The values of $\gamma$ are 57.1 ± 3.8, 67.0 ± 8.5, 73.4 ± 5.8, and 106 ± 15 pN/μm, respectively (displayed in Fig. 4b). They follow the same order as the zero-shear viscosities, and reflect successive strengthening of macromolecular networks. The ratio of interfacial tensions between the last member (S:L) and the first member (pK:H) of the series is 1.9. The droplet stretching method for interfacial tension was validated by measuring the force required to rupture the droplet surface when a trapped bead was pulled from inside (Fig. 4b; Supplementary Movie 3 and Supplementary Fig. 6). The material properties that affect the fusion times of the condensates are collected in Table 1 for easy comparison.

**Table 1:** Material properties and fusion times of four types of condensates.

	$\eta$ (Pa s)	$\gamma$ (pN/μm)	${\tau }_{1}$ (ms)	${\tau }_{{{{{{\rm{fu}}}}}}}^{\ast }\,$(ms)	${\tau }_{1}/{\tau }_{{{{{{\rm{fu}}}}}}}^{\ast }$	${\tau }_{{{{{{\rm{fu}}}}}}}$ (ms)	${\tau }_{{{{{{\rm{fu}}}}}}}^{{{{{{\rm{N}}}}}}}$ (ms)	% diff
pK:H	0.30 ± 0.03	57.1 ± 3.8	45 ± 11	31	1.5	30.3 ± 0.9	31.4	3.4
P:H	0.53 ± 0.04	67.0 ± 8.5	125 ± 14	43	2.9	39.3 ± 3.9	47.1	18
S:P	3.75 ± 0.14	73.4 ± 5.8	72 ± 3	340	0.21	384 ± 31	302	24
S:L	10.1 ± 1.1	106 ± 15	396 ± 187	996	0.40	1774 ± 128	559	104

With both $\eta$ and $\gamma$ at hand, we can predict the fusion time of two equal-sized droplets (with radius $R$) if condensates inside are Newtonian fluids. This is given by^26^

3 $${\tau }_{{{{{{\rm{fu}}}}}}}^{{{{{{\rm{N}}}}}}}=1.97\frac{\eta R}{\gamma }=1.97{\tau }_{{{{{{\rm{vc}}}}}}}$$

Our measurements of fusion times (${\tau }_{{{{{{\rm{fu}}}}}}}$) were done on droplets with $R$ in the range of 0.8 to 8 μm, and established a proportional relation between ${\tau }_{{{{{{\rm{fu}}}}}}}$ and $R$ (here and in ref. ^26^). Because the zero-shear viscosities of the four types of droplets vary by 33-fold while the interfacial tensions vary by 1.9-fold, Eq. 3 predicts an 18-fold difference in fusion time between S:L and pK:H droplets. However, the measured fusion times, determined here for pK:H and reported previously for the other three types of droplets^26^, vary by 59-fold for the same $R$. The measured and predicted fusion times, both at $R$ = 3 μm, are compared for each type of droplets in Fig. 4c and in Table 1. The percentage differences between measured ${\tau }_{{{{{{\rm{fu}}}}}}}$ and predicted ${\tau }_{{{{{{\rm{fu}}}}}}}^{{{{{{\rm{N}}}}}}}$ are 3.4, 18, 24, and 104%, respectively, for pK:H, P:H, S:P, and S:L. The viscocapillary model overpredicts fusion times for the first two types of droplets but underpredicts them for the last two types of droplets. The opposite directions of the discrepancies for the first and last members of the series account for the much narrower range of the predicted fusion times.

## Discussion

It is thus clear that the four types of condensates exhibit non-Newtonian behaviors to varying extents during droplet fusion. S:P and S:L condensates deviate from Newtonian fluids in one direction (${\tau }_{{{{{{\rm{fu}}}}}}} > {\tau }_{{{{{{\rm{fu}}}}}}}^{{{{{{\rm{N}}}}}}}$) but pK:H and P:H condensates deviate from Newtonian fluids in the opposite direction (${\tau }_{{{{{{\rm{fu}}}}}}} < {\tau }_{{{{{{\rm{fu}}}}}}}^{{{{{{\rm{N}}}}}}}$). The opposite non-Newtonian behaviors indicate shear thickening (increase in viscosity) and shear thinning (decrease in viscosity), respectively. At increasing shear rate (i.e., rate of relative deformation; denoted by $\dot{\widetilde{{{{{{\rm{\varepsilon }}}}}}}}$), associative polymer solutions generally exhibit three regimes: Newtonian behavior at low $\dot{\widetilde{{{{{{\rm{\varepsilon }}}}}}}}{\tau }_{1}$; shear thickening as $\dot{\widetilde{{{{{{\rm{\varepsilon }}}}}}}}{\tau }_{1}$ increases toward 1; and shear thinning at even higher $\dot{\widetilde{{{{{{\rm{\varepsilon }}}}}}}}{\tau }_{1}$ (e.g., ref. ^36^; see also Supplementary Text). For droplet fusion, $\dot{\widetilde{{{{{{\rm{\varepsilon }}}}}}}}$ is measured by $1/{\tau }_{{{{{{\rm{fu}}}}}}}^{\ast }$ (relative deformation of order 1 during time ${\tau }_{{{{{{\rm{fu}}}}}}}^{\ast }\equiv {({\tau }_{{{{{{\rm{fu}}}}}}}{\tau }_{{{{{{\rm{fu}}}}}}}^{{{{{{\rm{N}}}}}}})}^{1/2}$). We thus expect the effective viscosity (${\eta }_{{{{{{\rm{eff}}}}}}}$) to have a dependence on ${\tau }_{1}/{\tau }_{{{{{{\rm{fu}}}}}}}^{\ast }$ that is characteristic of associative polymer solutions (orange curve in Fig. 4d). S:P condensates exhibit moderate shear thickening (${\tau }_{{{{{{\rm{fu}}}}}}}/{\tau }_{{{{{{\rm{fu}}}}}}}^{{{{{{\rm{N}}}}}}}\equiv {\eta }_{{{{{{\rm{eff}}}}}}}/\eta$ = 1.27), because their ${\tau }_{1}$ (= 0.21${\tau }_{{{{{{\rm{fu}}}}}}}^{\ast }$) is short relative to the fusion time. S:L condensates have a longer ${\tau }_{1}$ (= 0.46${\tau }_{{{{{{\rm{fu}}}}}}}^{\ast }$) and thus exhibit significant shear thickening (${\eta }_{{{{{{\rm{eff}}}}}}}/\eta$ = 3.17), which arises from shear-induced conversion of loose association into close association (Fig. 4d, illustration at the top). Both pK:H and P:H condensates have reconfiguration times longer than their respective fusion times. For pK:H, ${\tau }_{1}/{\tau }_{{{{{{\rm{fu}}}}}}}^{\ast }$ = 1.5 and ${\eta }_{{{{{{\rm{eff}}}}}}}/\eta$ = 0.97, corresponding to a point where shear-thickening has just crossed over to shear thinning. On the other hand, P:H has a much longer relative ${\tau }_{1}$ (= 2.9${\tau }_{{{{{{\rm{fu}}}}}}}^{\ast }$), and thus exhibits stronger shear thinning (${\eta }_{{{{{{\rm{eff}}}}}}}/\eta$ = 0.83), due to shear-induced breakup of interchain bridges (Fig. 4d, illustration on the right). We hope that our work will stimulate other experimental as well as theoretical studies to test these proposed explanations.

The present work reveals the shear relaxation time, ${\tau }_{1}$, as a key determinant of condensate fusion dynamics. For our condensates, ${\tau }_{1}$ ranges from 45 ms for pK:H to 396 ms for S:L. Alshareedah et al.^32^ reported a 60 ms relaxation time for droplets formed by mixing an RG-repeat peptide (RGRGG_5_) with polyuridine, and an increase to 900 ms upon substituting the middle R with a Y. For PGL-3 protein droplets, the relaxation time of fresh samples were < 10 ms^10,31^. Interestingly, over the course of 45 h the relaxation time increased to 11 min, indicating that a possible characteristic of condensate aging is a significant slowdown in shear relaxation^10^. Measurements of the complex shear moduli, ${G}^{\ast }\left(\omega \right)$, for more condensates, along with theoretical studies, will be required to unravel the molecular basis of the shear relaxation time.

The zero-shear viscosities of the four types of condensates studied here extend from 0.30 Pa s for pK:H to 10.1 Pa s for S:L. These values fall within the range reported in the literature for in vitro biomolecular condensates^6,20,21,27,29,37–39^, from 0.1 Pa s (PGL-3 droplets^29,31^ and droplets formed by mixing pK with uridine triphosphate^42^) to ~200 Pa s (GAR-1ΔN protein droplets^37^ and droplets formed by mixing polyarginine with polyuridine^42^). The interfacial tensions of our condensates range from 57.1 pN/μm for pK:H to 106 pN/μm for S:L. There are fewer direct measurements of interfacial tension in the literature, with values as low as ~1 pN/μm for PGL-3 droplets^29,31^ and as high as ~100 pN/μm for nucleophosmin protein droplets^6^ and FUS protein droplets^43^. Interfacial tensions have often been deduced by applying the viscocapillary model (Eq. 3), i.e., by combining fusion time with zero-shear viscosity. The present work makes it clear that this procedure is unreliable, as it fails to account for viscoelasticity and the consequent shear thickening or thinning of biomolecular condensates.

The condensates studied here are the ones for which data for viscoelasticity and interfacial tension (present work) as well as for fusion speed (present work and ref. ^26^) are available on the same system. This has put us in a unique position to test the viscocapillary model and the outcome calls the model into question. The four condensates studied here were originally chosen to exemplify the varied structural order of biomolecules of in vivo condensates. Now our data show that they fall into two groups. The macromolecular components in pK:H and P:H are fully disordered polymer chains, have lower viscosity, and exhibit shear-thinning during fusion. On the other hand, S:P and S:L contain structured domains, have higher viscosity, and exhibit shear thickening during fusion. Compared to polymer chains, interactions of structured domains result in closer macromolecular packing and higher packing densities (Fig. 3b), thereby explaining the higher viscosities of S:P and S:L. General correlates of molecular structural properties will emerge only after many more condensates are studied. Of our four condensates, three (pK:H, P:H, and S:L) were not studied by others, though pK:H is similar to a mixture of pK and polyuridine (highly negatively charged like H), for which zero-shear viscosity has been reported^42^. S:P is the only condensate studied by both us and others, and those studies concentrated on the determination of phase equilibrium, revealing the effects of multivalency^44^, inter-domain linkers^45^, appended disordered sequences^46^, and addition of macromolecular regulators^47^. A recent computational study has predicted that the effects on phase equilibrium can match those on interfacial tension^48^. The methods for measuring interfacial tension presented here will allow a test of this prediction.

We have demonstrated that diverse condensates are viscoelastic on the ms to s timescales. During fusion, condensates can deviate from Newtonian fluids in opposite directions (i.e., shear thickening or thinning), depending on the relative rates of shear relaxation and condensate deformation. The main mode of shear relaxation inside condensates is reconfiguration of macromolecular networks. While determination of phase equilibrium (and interfacial tension) yields information on energetic properties of macromolecular components, measurement of shear relaxation moduli enables us to probe dynamic properties of these molecules. Similar to fusion, other condensate dynamic processes, including shape recovery after deformation, physical association of different condensates, multiphase organization, assembly and disassembly, and aging, all involve reconfiguration of macromolecular networks. Shear relaxation provides the governing measure of condensate dynamics.

## Methods

### Sample preparation

Preparation of macromolecular droplets containing polystyrene beads for optical trapping was modified from a procedure in a previous study on fusion dynamics ^26^. Pentameric constructs of SH3 domains (S) and proline-rich motifs (P) were expressed and purified as reported, as was the labeling of S to produce Alexa 594-S ^47^. The sources for L, H, FITC-heparin, pK, and Ficoll70 were the same as reported ^41,47^. Carboxylate-coated polystyrene beads (2 µm diameter) were procured from Polysciences Inc. (Catalog# 18327-10). The working buffer was 10 mM imidazole pH 7 with 0.01% (w/v) NaN3 and KCl at 0.15 M unless otherwise indicated. Droplet samples containing beads were prepared by sequentially adding buffer, KCl if needed, pre-diluted bead solution, pre-dissolved Ficoll70, the negatively charged macromolecular component, and finally the positively charged component. The final macromolecular concentrations (in µM) were: pK:H = 100:100; P:H = 40:40; S:P = 40:40; and S:L = 20:300; the Ficoll70 concentration was 50 g/L. For pK:H droplets, the KCl concentration was 1.0 M; P:H droplets were prepared at 0.15, 0.3, and 0.4 M KCl. These samples matched in composition with those the study on fusion dynamics ^26^, except for the doubling of the pK and H concentrations in the pK:H samples and the presence of beads, which were at a concentration of ~0.01% (w/v). All sample preparations and OT based experiments were done at room temperature.

### Calibration of optical trap stiffness

The stiffnesses of the traps in a LUMICKS C-TrapTM dual-trap OT instrument were calibrated by fitting the power spectrum of a trapped bead’s position to that of a Brownian harmonic oscillator. Bead samples for calibration were diluted to a concentration of ~0.01 % (w/v) in deionized water. The fitting of the power spectrum yielded the corner frequency ${\omega }_{{{{{{\rm{c}}}}}}}/2\pi$, which is related to the stiffness, ${\kappa }_{{{{{{\rm{t}}}}}}}$, of the trap via

4 $${\omega }_{{{{{{\rm{c}}}}}}}=\frac{{\kappa }_{{{{{{\rm{t}}}}}}}}{{\xi }_{{{{{{\rm{m}}}}}}0}}$$

The remaining symbol, ${\xi }_{{{{{{\rm{m}}}}}}0}$ denotes the friction coefficient of the bead in the surrounding medium, and is given by ${\xi }_{{{{{{\rm{m}}}}}}0}=6\pi \eta a$, where $\eta$ is the (zero-shear) viscosity of the medium and $a$ is the radius of the trapped bead. In short, the trap stiffness is determined by measuring the corner frequency and entering the medium viscosity and the bead radius. However, ${\kappa }_{{{{{{\rm{t}}}}}}}$ is unaffected by the medium viscosity.

The trap stiffness ${\kappa }_{{{{{{\rm{t}}}}}}}$ depends on the refractive index of the medium, and hence its value inside macromolecular droplets could potentially differ from that calibrated in deionized water. However, Jawerth et al.^29^ have demonstrated in their experiments that the trap stiffness is “largely unchanged by the presence of the droplet within the measurement error.” Likewise, Fischer et al.^49^ found the trap stiffnesses inside water and inside an F-actin network to be within errors of each other. These observations can be rationalized by noting that the protein solute has only a very small effect on the refractive index. Measurements of refractive index (at 578 nm wavelength) in concentrated protein solutions found a refraction increment of 0.000183*C*, where *C* is protein concentration in mg/mL^50^. For S:P droplets, we previously determined the S concentration to be ~1 mM^47^. This concentration measurement entailed careful determination of standard curves and avoided the errors arising from equating fluorescence intensity ratios to protein concentration ratios. Assuming an equal molar concentration for P, the total macromolecular concentration inside S:P droplets can be estimated to be 56 mg/mL, corresponding to a refraction increment of merely 0.01. The refractive index inside S:P droplets was not measured, but the above estimation gives a value of 1.34, compared to 1.33 in water. Therefore it is entirely reasonable for the trap stiffness calibrated in water to be applicable inside macromolecular droplets.

To unequivocally verify that the trap stiffness is largely unchanged inside the macromolecular droplets studied here, we determined the trap stiffnesses directly inside the droplets by an active-passive calibration procedure. The idea for this procedure originated from Fischer and Berg-Sorensen^51^. The passive part of the calibration involves acquiring the power spectrum of the position of a bead optically trapped inside the working medium (e.g., water or the interior of a macromolecular droplet). The movement of the bead position $x$ relative to the fixed optical trap obeys Newton’s equation:

5 $$m\ddot{x}=-{\xi }_{{{{{{\rm{m}}}}}}}\dot{x}-{\kappa }_{{{{{{\rm{m}}}}}}}x-{\kappa }_{{{{{{\rm{t}}}}}}}x{{{{{\mathscr{+}}}}}}{{{{{\mathcal{R}}}}}}$$

where $m$ is the bead mass, ${\xi }_{{{{{{\rm{m}}}}}}}$ is the friction coefficient of the medium (proportional to the viscous modulus), ${\kappa }_{{{{{{\rm{m}}}}}}}$ is the spring constant of the medium (proportional to the elastic modulus), ${\kappa }_{{{{{{\rm{t}}}}}}}$ is the stiffness of the optical trap, and ${{{{{\mathscr{R}}}}}}$ is a random force. From a time trace of the bead position, we can calculate the autocorrelation function

6 $${C}_{x}(t)=\frac{1}{{t}_{{{{{{\rm{msr}}}}}}}}\int_{-\frac{{t}_{{{{{{\rm{msr}}}}}}}}{2}}^{\frac{{t}_{{{{{{\rm{msr}}}}}}}}{2}} dt^{{\prime}} x\left({t}^{{\prime} }+t\right)x(t^{\prime} )$$

where ${t}_{{{{{{\rm{msr}}}}}}}$ is the total time over which the bead position is measured. Note that ${C}_{x}(0)$ is the mean-squared displacement of the bead, which according to the equipartition theorem is given by

7 $${C}_{x}(0)=\frac{{k}_{{{{{{\rm{B}}}}}}}T}{{\kappa }_{{{{{{\rm{t}}}}}}}}$$

where *k*_B_ is the Boltzmann constant and *T* is the absolute temperature. The Fourier transform of ${C}_{x}(t)$ is the power spectrum, which, by taking the Fourier transform of Eq. 5, is found to be

8 $${P}_{x}(\omega )=\frac{2{k}_{{{{{{\rm{B}}}}}}}T{\xi }_{{{{{{\rm{m}}}}}}}(\omega )}{{{{{{{\rm{|}}}}}}{\kappa }_{{{{{{\rm{t}}}}}}}+{\kappa }_{{{{{{\rm{m}}}}}}}\left(\omega \right)+i\omega {\xi }_{{{{{{\rm{m}}}}}}}\left(\omega \right)-{\omega }^{2}m{{{{{\rm{|}}}}}}}^{2}}$$

where $\omega$ denotes the angular frequency (its division by $2\pi$ gives the frequency in Hz). At zero frequency, ${\kappa }_{{{{{{\rm{m}}}}}}}\left(0\right)$ = 0, ${\xi }_{{{{{{\rm{m}}}}}}}\left(0\right)={\xi }_{{{{{{\rm{m}}}}}}0}$, and Eq. 8 reduces to

9 $${P}_{x}(0)=\frac{2{k}_{{{{{{\rm{B}}}}}}}T}{{\kappa }_{{{{{{\rm{t}}}}}}}{\omega }_{{{{{{\rm{c}}}}}}}}$$

where ${\omega }_{{{{{{\rm{c}}}}}}}$ is given by Eq. 4. At sufficiently low frequencies, ${\kappa }_{{{{{{\rm{m}}}}}}}\left(\omega \right)$ and ${\omega }^{2}m$ can be neglected relative to ${\kappa }_{{{{{{\rm{t}}}}}}}$ while ${\xi }_{{{{{{\rm{m}}}}}}}(\omega )$ can be replaced by ${\xi }_{{{{{{\rm{m}}}}}}0}$, leading to

10 $${P}_{x}(\omega )\approx \frac{2{k}_{{{{{{\rm{B}}}}}}}T{\omega }_{{{{{{\rm{c}}}}}}}/{\kappa }_{{{{{{\rm{t}}}}}}}}{{\omega }^{2}+{\omega }_{{{{{{\rm{c}}}}}}}^{2}}=\frac{{P}_{x}(0)}{{(\omega /{\omega }_{{{{{{\rm{c}}}}}}})}^{2}+1}$$

This Lorentzian function is the power spectrum of a Brownian harmonic oscillator.

To make use of Eq. 7, we note that ${C}_{x}(0)$ is given by the integral of ${P}_{x}(\omega )$:

11 $${C}_{x}\left(0\right)=\frac{1}{\pi }\int_{0}^{{{\infty }}}d\omega {P}_{x}(\omega )=\frac{{k}_{{{{{{\rm{B}}}}}}}T}{{\kappa }_{{{{{{\rm{t}}}}}}}}$$

The integral of the power spectrum thus allows the determination of the trap stiffness. However, the bead displacement is reported by a quadrant photodiode in volts, not μm. Correspondingly, the raw power spectrum, ${P}_{{{{{{\rm{V}}}}}}}(\omega )$, is in units of V^2^/Hz. The conversion factor, $\epsilon$, from volt to μm also needs to be calibrated. This task is accomplished by the active part of the calibration. Applying the conversion factor, we have

12 $${P}_{x}\left(\omega \right)={\epsilon }^{2}{P}_{{{{{{\rm{V}}}}}}}\left(\omega \right)$$

Instead of directly integrating the power spectrum, we first fit it to a function. Here we choose a “stretched” Lorentzian function

13 $${P}_{{{{{{\rm{V}}}}}}}\left(\omega \right)=\frac{{P}_{{{{{{\rm{V}}}}}}}(0)}{{(\omega /{\omega }_{{{{{{\rm{eff}}}}}}})}^{\alpha }+1}$$

Integrating this function, Eq. 11 becomes

14 $${\kappa }_{{{{{{\rm{t}}}}}}}{\epsilon }^{2}=\frac{\alpha {{{{{\rm{sin }}}}}}(\pi /\alpha ){k}_{{{{{{\rm{B}}}}}}}T}{{\omega }_{{{{{{\rm{eff}}}}}}}{P}_{{{{{{\rm{V}}}}}}}(0)}$$

This is the first identity for calibration.

In the active part of the calibration, the optical trap is not fixed but driven in a sinusoidal motion. Note that this part of the calibration is exactly the same as the procedure for measuring the viscoelasticity (see subsection “Data analysis for bead oscillation”), except here we limit to low driving frequencies. The trap position can be expressed as

15 $${X}_{{{{{{\rm{t}}}}}}}(t)={X}_{{{{{{\rm{t}}}}}}0}{e}^{i(\omega t+\phi )}$$

where ${X}_{{{{{{\rm{t}}}}}}0}$ denotes the amplitude and $\phi$ denotes the phase angle at $t$ = 0. The interest now is $X,$ the bead position $x$ after averaging over thermal noise. Replacing $x$ in the term $-{\kappa }_{{{{{{\rm{t}}}}}}}x$ in Eq. 5 by $x-{X}_{{{{{{\rm{t}}}}}}}$ and averaging over thermal noise, we obtain

16 $$m\ddot{X}=-{\xi }_{{{{{{\rm{m}}}}}}}\dot{X}-{\kappa }_{{{{{{\rm{m}}}}}}}X-{\kappa }_{{{{{{\rm{t}}}}}}}\left[X-{X}_{{{{{{\rm{t}}}}}}}(t)\right]$$

Taking the Fourier transform of Eq. 16 and denoting Fourier transforms by the caret symbol, we find

17 $$\frac{\hat{X}(\omega )}{{\hat{X}}_{t}(\omega )}=\frac{{\kappa }_{{{{{{\rm{t}}}}}}}}{{\kappa }_{{{{{{\rm{t}}}}}}}+{\kappa }_{{{{{{\rm{m}}}}}}}\left(\omega \right)+i\omega {\xi }_{{{{{{\rm{m}}}}}}}\left(\omega \right)-{\omega }^{2}m}$$

The imaginary part of this expression has a simple relation to the power spectrum of Eq. 8:

18a $$-{{{{{\rm{Im}}}}}}\left[\frac{\hat{X}(\omega )}{{\hat{X}}_{t}(\omega )}\right]=\frac{{\kappa }_{{{{{{\rm{t}}}}}}}\omega {\xi }_{{{{{{\rm{m}}}}}}}\left(\omega \right)}{{{{{{{\rm{|}}}}}}{\kappa }_{{{{{{\rm{t}}}}}}}+{\kappa }_{{{{{{\rm{m}}}}}}}\left(\omega \right)+i\omega {\xi }_{{{{{{\rm{m}}}}}}}\left(\omega \right)-{\omega }^{2}m{{{{{\rm{|}}}}}}}^{2}}$$

18b $$=\frac{{\kappa }_{{{{{{\rm{t}}}}}}}\omega {P}_{x}(\omega )}{2{k}_{{{{{{\rm{B}}}}}}}T}\quad\qquad\;$$

This relation between $\hat{X}(\omega )$ and ${P}_{x}(\omega )$ is an example of Onsager’s regression hypothesis^52^, which states that “the average regression of fluctuations will obey the same laws as the corresponding macroscopic irreversible processes.” In the subsection “Data analysis for bead oscillation”, we show that

19 $$-{{{{{\rm{Im}}}}}}\left[\frac{\hat{X}\left(\omega \right)}{{\hat{X}}_{t}\left(\omega \right)}\right]=\frac{{F}_{{{{{{\rm{t}}}}}}0}{{{{{\rm{sin }}}}}}\triangle }{{\kappa }_{{{{{{\rm{t}}}}}}}{X}_{{{{{{\rm{t}}}}}}0}}$$

where ${F}_{{{{{{\rm{t}}}}}}0}$ is the ampltidue of the trapping force, and $\triangle$ is the phase difference of the trapping force from the trap position. Combining Eqs. 18b and 19, we have

20 $$\frac{{F}_{{{{{{\rm{t}}}}}}0}{{{{{\rm{sin }}}}}}\triangle }{{\kappa }_{{{{{{\rm{t}}}}}}}{X}_{{{{{{\rm{t}}}}}}0}}=\frac{{\kappa }_{{{{{{\rm{t}}}}}}}\omega {P}_{x}(\omega )}{2{k}_{{{{{{\rm{B}}}}}}}T}$$

The raw force, ${F}_{{{{{{\rm{V}}}}}}}$, is actually the bead displacement from the trap, in volts. The conversion to force in pN requires applying two factors, $\epsilon$ (from volt to μm) and ${\kappa }_{{{{{{\rm{t}}}}}}}$ (from μm to pN):

21 $${F}_{{{{{{\rm{t}}}}}}}={\kappa }_{{{{{{\rm{t}}}}}}}\epsilon {F}_{{{{{{\rm{V}}}}}}}$$

Inserting Eqs. 12 and 21 into Eq. 20, we find

22 $${\kappa }_{{{{{{\rm{t}}}}}}}\epsilon =\frac{2{k}_{{{{{{\rm{B}}}}}}}T}{{P}_{{{{{{\rm{V}}}}}}}\left(\omega \right)}\frac{{F}_{{{{{{\rm{V}}}}}}0}{{{{{\rm{sin }}}}}}\triangle }{\omega {X}_{{{{{{\rm{t}}}}}}0}}$$

where ${F}_{{{{{{\rm{V}}}}}}0}$ is the amplitude of the trapping force in volts. This is the second identity for calibration. Combining Eqs. 14 and 22 leads to the expression for calibrating $\epsilon$:

23a $$\epsilon =\frac{(\alpha /2){{{{{\rm{sin }}}}}}(\pi /\alpha ){P}_{{{{{{\rm{V}}}}}}}\left(\omega \right)}{{\omega }_{{{{{{\rm{eff}}}}}}}{P}_{{{{{{\rm{V}}}}}}}(0)}\frac{\omega {X}_{{{{{{\rm{t}}}}}}0}}{{F}_{{{{{{\rm{V}}}}}}0}{{{{{\rm{sin }}}}}}\triangle }$$

Using Eq. 13, we simplify the above expression to

23b $$\epsilon =\frac{(\alpha /2){{{{{\rm{sin }}}}}}(\pi /\alpha )\omega /{\omega }_{{\omega }_{{{{{{\rm{eff}}}}}}}}}{{(\omega /{\omega }_{{{{{{\rm{eff}}}}}}})}^{\alpha }+1}\frac{{X}_{{{{{{\rm{t}}}}}}0}}{{F}_{{{{{{\rm{V}}}}}}0}{{{{{\rm{sin }}}}}}\triangle }$$

Contrary to the calibration of Blehm et al.^53^, which relied on a detection laser in addition to the trapping laser, calibration using Eq. 23b does not require a second laser, nor does it require a piezo stage (as was the case in Fisher et al.^49^). Our active-passive calibration is unique by solely using the trapping laser.

We first verified the active-passive calibration procedure in water. Supplementary Fig. 3a presents the power spectrum of a bead trapped in water. As expected, it fits well to the Lorentzian function of a Brownian harmonic oscillator. The resulting corner frequency (3273 Hz) allows the determination of the trap stiffness when the bead radius (1 µm) and water viscosity are used. We drove the same bead in a sinusoidal motion at 1 Hz and obtained the time trace of the trapping force (Supplementary Fig. 3b). This active-passive calibration was done on four beads. The stiffness determined was 307 ± 33 pN. In comparison, the stiffness was found to be 307 ± 13 pN when the bead radius and water viscosity were used in lieu of active calibration.

The active-passive calibration was also carried out in macromolecular droplets. Supplementary Fig. 3c shows the power spectrum of a bead trapped inside a pK:H droplet, as well as the fit to a stretched Lorentzian function (Eq. 13). The power spectrum is noisier compared to the counterpart in water, as expected for a complex environment. Moreover, ${\omega }_{{{{{{\rm{eff}}}}}}}/2\pi$ is now below 10 Hz, which can be attributed to the much higher viscosity inside the droplet. Again, we drove the same bead in a sinusoidal motion, at 0.1 Hz, and obtained the time trace of the trapping force (Supplementary Fig. 3d). This active-passive calibration was done on seven beads. The stiffness determined was 316 ± 17 pN, which agrees with the counterpart determined in water to within measurement errors.

### Oscillation of a trapped bead inside settled droplets

Carboxylate-coated beads inside settled droplets were oscillated at amplitudes of 1.0 to 0.25 μm and frequencies ($\omega /2\pi$) of 0.5 to 40 Hz by the LUMICKS C-Trap^TM^ instrument (Fig. 2a). Following the preparation of bead-containing droplets, a 7–8 µL aliquot was loaded onto a custom-made sample chamber^26^, and droplets were allowed (for ~10 min) to settle on the coverslip. The power of the trapping laser (100% given to trap 1) was then turned up sufficiently to trap a bead inside a settled droplet and oscillate the bead at a chosen amplitude and frequency.

Only sufficiently large droplets were selected so that a centrally trapped bead would be at least several bead diameters away from any boundary. A brightfield image guided the positioning of the trapped bead at a transversely central location inside the droplet; further positioning at the droplet center along the longitudinal (i.e., *z*) direction was achieved when the trapped bead and the droplet equator were both in sharp focus. *z* positions that were either too high or too low (such that the bead got close to the top or bottom boundary) resulted in an in-phase trap position and force traces at the lowest frequency (i.e., 0.5 Hz). In contrast, a *z* position away from droplet boundaries resulted in a phase difference of >*π*/4 at 0.5 Hz.

A complete set of data was collected on each trapped bead and exported as hdf files containing the trap 1 *x* position traces in volts and the trap 1 force *x* component traces in pN over a few s, at frequencies from 0.5 to 40 Hz. Replicates (*N* = 3 or 4) were performed on different droplets from the same sample or from different samples.

### Data analysis for bead oscillation

An optically trapped bead inside viscoelastic fluids experiences three forces^33,34^: a viscous force proportional to the velocity $\dot{X}$ of the bead, an elastic force proportional to the displacement $X$ of the bead, and the trapping force ${F}_{{{{{{\rm{t}}}}}}}$ proportional to the displacement, $X-{X}_{{{{{{\rm{t}}}}}}}$, between the bead and trap. Neglecting inertial effects, the governing Eq. 16 becomes

24 $$-{\xi }_{{{{{{\rm{m}}}}}}}\dot{X}-{\kappa }_{{{{{{\rm{m}}}}}}}X-{\kappa }_{{{{{{\rm{t}}}}}}}(X-{X}_{{{{{{\rm{t}}}}}}})=0$$

When the trap is driven in a sinusoidal motion, with the position given by Eq. 15, the trapping force can be expressed as

25 $${F}_{{{{{{\rm{t}}}}}}}=-{\kappa }_{{{{{{\rm{t}}}}}}}\left(X-{X}_{{{{{{\rm{t}}}}}}}\right)={F}_{{{{{{\rm{t}}}}}}0}{e}^{i(\omega t+\phi +\triangle )}$$

From the last two equations, we can find the bead position as

26 $$X={X}_{0}{e}^{i(\omega t+\phi -\delta )}$$

where

27 $${X}_{0}{e}^{-i\delta }={X}_{{{{{{\rm{t}}}}}}0}-\frac{{F}_{{{{{{\rm{t}}}}}}0}}{{\kappa }_{{{{{{\rm{t}}}}}}}}{e}^{i\triangle }$$

The imaginary parts of this equation lead to Eq. 19, upon recognizing that $\hat{X}(\omega )/{\hat{X}}_{t}(\omega )={X}_{0}{e}^{-i\delta }/{X}_{{{{{{\rm{t}}}}}}0}$. Using Eqs. 25 and 26 in Eq. 24, we find

28 $$\left({\kappa }_{{{{{{\rm{m}}}}}}}+i\omega {\xi }_{{{{{{\rm{m}}}}}}}\right){X}_{0}{e}^{-i\delta }={F}_{{{{{{\rm{t}}}}}}0}{e}^{i\triangle }$$

The quantity, ${\kappa }_{{{{{{\rm{m}}}}}}}+i\omega {\xi }_{{{{{{\rm{m}}}}}}}$, in the parentheses defines the complex shear modulus ${G}^{\ast }(\omega )$,

29 $$6\pi a{G}^{\ast }(\omega )={\kappa }_{{{{{{\rm{m}}}}}}}+i\omega {\xi }_{{{{{{\rm{m}}}}}}}$$

where $a$ is the radius of the trapped bead (1 μm in our case). Rearranging Eq. 28, we find

30 $${G}^{\ast }\left(\omega \right)\equiv {G}^{{\prime} }\left(\omega \right)+{iG}{{{{{\rm{\hbox{''}}}}}}}(\omega )=\frac{{F}_{{{{{{\rm{t}}}}}}0}{e}^{i\triangle }}{6\pi a{X}_{0}{e}^{-i\delta }}=\frac{{F}_{{{{{{\rm{t}}}}}}0}}{6\pi a{X}_{{{{{{\rm{t}}}}}}0}}\frac{{e}^{i\triangle }}{1-{{{{{\rm{{\Upsilon }}}}}}}{e}^{i\triangle }}$$

where in the last step we have used Eq. 27, and ${{{{{\rm{{\Upsilon }}}}}}}={F}_{{{{{{\rm{t}}}}}}0}/{\kappa }_{{{{{{\rm{t}}}}}}}{X}_{{{{{{\rm{t}}}}}}0}$. The real and imaginary parts, ${G}^{{\prime} }\left(\omega \right)$ and $G\mbox{''}(\omega )$, are the elastic and viscous moduli, respectively. The final expressions for them are

31a $$G^{\prime} (\omega )=\frac{{F}_{{{{{{\rm{t}}}}}}0}}{6\pi a{X}_{{{{{{\rm{t}}}}}}0}}\frac{{{{{{\rm{cos }}}}}}\triangle -{{{{{\rm{{\Upsilon }}}}}}}}{{({{{{{\rm{cos }}}}}}\triangle -{{{{{\rm{{\Upsilon }}}}}}})}^{2}+{{{{{{\rm{sin }}}}}}}^{2}\triangle }$$

31b $$G{{{{{\rm{\hbox{''}}}}}}}(\omega )=\frac{{F}_{{{{{{\rm{t}}}}}}0}}{6\pi a{X}_{{{{{{\rm{t}}}}}}0}}\frac{{{{{{\rm{sin }}}}}}\triangle }{{({{{{{\rm{cos }}}}}}\triangle -{{{{{\rm{{\Upsilon }}}}}}})}^{2}+{{{{{{\rm{sin }}}}}}}^{2}\triangle }$$

The trap position and force traces from the bead oscillation experiments were analyzed using MATLAB scripts. Both signals were fit to a cosine wave function (Fig. 2b):

32 $${X}_{{{{{{\rm{t}}}}}}}={X}_{{{{{{\rm{t}}}}}}0}{{{{{\rm{cos }}}}}}(\omega t+\phi )+{B}_{1}$$

33 $${F}_{{{{{{\rm{t}}}}}}}={F}_{{{{{{\rm{t}}}}}}0}{{{{{\rm{cos }}}}}}(\omega t+\phi +\triangle )+{B}_{2}$$

The resulting amplitudes ${X}_{{{{{{\rm{t}}}}}}0}$ and ${F}_{{{{{{\rm{t}}}}}}0}$ and the phase difference $\triangle$, along with the trap stiffness ${\kappa }_{{{{{{\rm{t}}}}}}}$, were used in calculating the elastic and viscous moduli according to Eq. 31a, b. ${X}_{{{{{{\rm{t}}}}}}0}$ was converted to μm using a conversion factor from volt to μm, obtained by assuming that the trap position amplitude at 0.5 Hz was the preset value (e.g., 1.0 μm). The resulting ${X}_{{{{{{\rm{t}}}}}}0}$ values at the highest frequencies (close to and at 40 Hz) could be somewhat less than the preset values.

The foregoing experimental and data analysis protocols were validated by oscillating trapped beads (at frequencies between 0.5 and 10 Hz) in deionized water and glycerol solutions. This control experiment yielded the viscosity of the surrounding medium as $G\mbox{''}(\omega )/\omega$. The results were consistent with literature values.

### Stretching of droplets suspended by two trapped beads at the opposite poles

Droplets were suspended by two trapped beads at the opposite poles, in a configuration introduced by Jawerth et al.^29^ (Fig. 2c, top panel). As first proposed in ref. ^30^, the droplet was stretched to obtain its static spring constant ${\chi }_{0}$, which is proportional to the interfacial tension. Stretching is a more convenient protocol than the oscillatory one used by Jawerth et al., because it specifically and solely probes the interfacial tension.

To prepare the suspended configuration, all droplets and beads were first allowed to settle on the coverslip. Two beads were then trapped and lifted from the bottom. Around each lifted bead, a droplet grew. By bringing the two beads toward each, the droplets fused spontaneously to generate the suspended configuration, where the trapped beads are located at the opposite poles of the fused droplet. In cases where settled beads were difficult to lift due to high viscosity or interfacial tension (as found in S:L samples), beads were trapped before settling on the coverslip in order to generate the suspended configuration; the suspended droplet was then held until all neighboring beads and droplets had settled. From this configuration, trap 2 was fixed while trap 1 was pulled away at a very low speed (0.05 μm/s), for a total of close to 0.5 μm. Pulling speeds of 0.01 and 0.1 μm/s were also tested to verify that these pulling speeds had no effect on the forces measured. Moreover, stretching and subsequent retracting yielded the same ${\chi }_{0}$, showing that the droplets indeed behaved like a spring. The data for trap 1 position and traps 1 and 2 force *x* components over the entire pulling period were exported as hdf files. The brightfield images of the entire process were also recorded in video. Replicates (*N* = 10 to 13) were performed on different suspended droplets.

### Data analysis for droplet stretching

The static spring constant of a droplet is

34 $${\chi }_{0}=\frac{({F}_{{{{{{\rm{t}}}}}}2}-{F}_{{{{{{\rm{t}}}}}}1})/2}{\triangle X}$$

where ${F}_{{{{{{\rm{t}}}}}}1}$ and ${F}_{{{{{{\rm{t}}}}}}2}$ are the forces exerted by trap 1 and trap 2, respectively, at the opposite poles, and $\triangle X$ is the resulting stretch of the droplet. $\triangle X$ is measured by the change in the inter-bead distance. Instead of $\triangle X$, we directly tracked, $\triangle {X}_{{{{{{\rm{t}}}}}}1}$, the movement of trap 1 relative to trap 2, which was kept fixed (Fig. 2c, bottom panel). The quantity analogous to ${\chi }_{0}$ is

35 $${\chi }_{{{{{{\rm{sys}}}}}}0}=\frac{({F}_{{{{{{\rm{t}}}}}}2}-{F}_{{{{{{\rm{t}}}}}}1})/2}{-\triangle {X}_{{{{{{\rm{t}}}}}}1}}$$

The trapping forces are

36 $${F}_{{{{{{\rm{t}}}}}}j}=-{\kappa }_{{{{{{\rm{t}}}}}}j}\left({X}_{j}-{X}_{{{{{{\rm{t}}}}}}j}\right),j=1\; {{{{{\rm{and}}}}}}\; 2$$

Force balance on the system in suspension yields

37 $${F}_{{{{{{\rm{t}}}}}}1}+{F}_{{{{{{\rm{t}}}}}}2}=0$$

We can identify $\triangle X$ as ${X}_{2}-{X}_{1}-{d}_{0}$, where ${d}_{0}$ is the initial value of ${X}_{2}-{X}_{1}$. Note ${d}_{0}$ is also the initial value of ${X}_{{{{{{\rm{t}}}}}}2}-{X}_{{{{{{\rm{t}}}}}}1}$ since initially ${X}_{j}={X}_{{{{{{\rm{t}}}}}}j}$. Moreover, ${X}_{{{{{{\rm{t}}}}}}2}$ is held constant. Therefore $-\triangle {X}_{{{{{{\rm{t}}}}}}1}=$ ${X}_{{{{{{\rm{t}}}}}}2}-{X}_{{{{{{\rm{t}}}}}}1}-{d}_{0}$. Using these relations, we derive

38 $$\frac{1}{{\chi }_{0}}=\frac{1}{{\chi }_{{{{{{\rm{sys}}}}}}0}}-\frac{1}{{\kappa }_{{{{{{\rm{t}}}}}}1}}-\frac{1}{{\kappa }_{{{{{{\rm{t}}}}}}2}}$$

This result reflects the fact that the system containing the droplet and two optical traps is equivalent to three springs connected in series.

We verified that, at the end of each stretching experiment, trap 1 indeed moved the preset amount (0.5 μm; converted from trap 1 position in volts using the aforementioned conversion factor). The trap 1 position changed as a linear function of time, though the negative slope $v$, corresponding to the pulling speed, was somewhat less than the preset value (0.05 μm/s) as the pulling period was longer than the expected time of 10 s (see Fig. 2d). The trap 1 force *x* component and trap 2 force *x* component, after smoothing with moving average in a 64-ms window, were each fit to a linear function of time, with slopes ${f}_{1}$ and ${f}_{2}$. The static spring constant of the system comprising the droplet and two trapped beads was found as

39 $${\chi }_{{{{{{\rm{sys}}}}}}0}=\frac{({f}_{2}-{f}_{1})/2}{v}$$

This result along with the stiffnesses of the traps were then used to obtain the static spring constant ${\chi }_{0}$ of the droplet itself.

As shown previously^30^, ${\chi }_{0}$ is proportional to the interfacial tension $\gamma$ of the droplet:

40 $${\chi }_{0}=\frac{\pi \alpha ({\theta }_{0})}{2}\gamma$$

where ${\theta }_{0}$ is the polar angle spanned by the trapped bead at each pole and is ~$a/(R-a)$, where $R$ and $a$ are the radii of the unstretched droplet and the bead, respectively. For ${\theta }_{0}$ up to 0.5, $\alpha ({\theta }_{0})$ is accurately given by

41 $$\frac{1}{\alpha \left({\theta }_{0}\right)}=-0.5{{{{{\rm{ln}}}}}}{\theta }_{0}+0.34$$

The interfacial tension was thus found as

42 $$\gamma =\frac{1}{\pi }[{{{{{\rm{ln}}}}}}(R/a-1)+0.68]{\chi }_{0}$$

We determined the diameter ($2R$) of the suspended droplet by measuring the edge-to-edge distance of its image at the start of the recorded video using imageJ. The measurement was in pixels, which was converted to μm using the conversion factor of 0.0866 μm per pixel. Droplet diameters ranged from 7 to 14 μm.

### Surface rupture of settled droplets by a trapped bead

To validate the interfacial tensions measured by stretching droplets, we pulled a trapped bead inside as a settled droplet until the bead ruptured the droplet surface. Similar to a Wilhelmy plate^54^ or a du Noüy ring^55^, the trapped bead acted as a micro-tensiometer.

The initial setup was similar to what is described under “Oscillation of a trapped bead inside settled droplets”, except this time the trapped bead was lifted to near the top of the droplet, to ensure that the resulting surface protrusion did not adhere to the coverslip. The bead was pulled at a low constant speed (0.1 μm/s) until it ruptured the droplet surface. The trace of the trapping force was exported. Replicates (*N* = 14 to 20) were performed on different settled droplets.

Similar to the situation with a Wilhelmy plate or a du Noüy ring, the maximum trapping force ${F}_{{{{{{\rm{rup}}}}}}}$, corresponding to the moment where the droplet surface is ruptured by the bead, is related to the force due to the interfacial tension. This tension force can be estimated as the product of $\gamma$ and $2\pi a$, the circumference of the bead’s equator. Additionally, the bead is pulled back by intermolecular molecules between the coating carboxylates and the droplet macromolecular components. A crude assumption is that the latter force is proportional to the tension force. Force balance thus yields

43 $${F}_{{{{{{\rm{rup}}}}}}}=2\pi {af}\gamma$$

where $f$ is a numerical constant. The rupture forces for the four types of droplets studied here ranked in the same order as the interfacial tensions measured by the droplet stretching method. By choosing $f$ = 1.1, the interfacial tensions converted from rupture forces according to Eq. 43 are in quantitative agreement with the values from droplet stretching.

### Fusion speed of pK:H droplets at an equimolar concentration of 100 μM

We previously measured the fusion speed of pK:H droplets at an equimolar concentration of 50 μM^26^. At this concentration, the droplets were small, making it difficult to determine the viscoelasticity by oscillating a trapped bead inside. Larger droplets were formed when the component concentration was increased to 100 μM; hence we worked with this concentration in the present study. To ensure that viscoelasticity, interfacial tension, and fusion speed were also measured for exactly the same system, here we measured the fusion speed for pK:H droplets at 100 μM (in the presence of 50 g/L Ficoll70). The inverse fusion speed, measured on 17 droplets ranging 0.8 to 4 μm in radius, was 10.1 ± 0.3 ms/μm, compared to 6.7 ± 0.2 ms/μm found previously for pK:H droplets at 50 μM^26^.

### Reporting Summary

Further information on research design is available in the Nature Research Reporting Summary linked to this article.

## Supplementary information

Supplementary Information

Description of Additional Supplementary Files

Supplementary Movie 1

Supplementary Movie 2

Supplementary Movie 3

Reporting Summary

## Data Availability

The viscoelasticity data and interfacial tension data have been posted on GitHub at https://github.com/hzhou43/viscoelasticity-of-biomolecular-condensates/tree/main/Data. The data are in the folders Figs. 3a and 4b. Other data reported in Fig. 4a–c are listed in Table 1.

## Code availability

The custom codes in MATLAB (Version R2018b) for analyzing primary data on viscoelasticity and interfacial tension; GNUPlot (Version 5.0 patch level 3) script for fitting the frequency dependences of the elastic and viscous moduli to the Burgers model are available on Github https://github.com/hzhou43/viscoelasticity-of-biomolecular-condensates/tree/main/Codes.
