# Supplementary material for: Shear relaxation governs fusion dynamics of biomolecular condensates
Source: Nat Commun. 2021 Oct 13;12:5995. doi: 10.1038/s41467-021-26274-z (PMC8514506; doi:10.1038/s41467-021-26274-z)
Supplement: Supplementary file 1 — Supplementary Information [file 41467_2021_26274_MOESM1_ESM.pdf]

Supplementary Information for

Shear Relaxation Governs Fusion Dynamics of Biomolecular Condensates

Archishman Ghosh[1], Divya Kota[1], and Huan-Xiang Zhou[1,2]*

AG and DK contributed equally to this work.
Correspondence to: hzhou43@uic.edu

This PDF file includes:

Supplementary Text
Supplementary Figures 1 to 6
Supplementary References

Other Supplementary Materials for this manuscript include the following:

Supplementary Movies 1 to 3

Supplementary Text

Viscous liquids vs. elastic solids: distinction in shear relaxation

In a viscous liquid, a bead experiences a frictional force that is proportional to its velocity. In contrast, in an elastic solid, the bead experiences a resistance that is proportional to its displacement. A corresponding contrast between the viscous liquid and elastic solid exists when these materials are deformed by shearing. The resulting stress, $\tilde{\tau}$, is proportional to the shear rate $\dot{\tilde{\varepsilon}} = \frac{\partial}{\partial t}\tilde{\varepsilon}$ in the liquid but is proportional to the shear strain $\tilde{\varepsilon}$ itself in the solid.

Viscous liquids and elastic solids are opposite extremes of viscoelastic fluids. The latter materials, including biomolecular condensates, generally behave as partly liquid and partly solid. There the stress is determined by the entire history of the shear rate:

$$\tilde{\tau}(t) = \int_{-\infty}^{t} dt' G(t - t')\,\dot{\tilde{\varepsilon}}(t') \qquad \text{[S1]}$$

The function $G(t)$ is called the shear relaxation modulus. This expression for the shear stress is similar in form and substance to the frictional force on a generalized Langevin particle; the counterpart of $G(t - t')$ is the memory kernel. Note that $G(t - t')$ must be 0 when $t' > t$ (such that future shear rate does not affect present stress); hence the upper limit of the integral can extend to $+\infty$. In a purely viscous liquid (also known as a Newtonian fluid), the stress is affected by the shear rate only at the present time, not any earlier time. That is,

$$\tilde{\tau}(t) = \eta\dot{\tilde{\varepsilon}}(t) \qquad \text{[S2a]}$$

where η is the viscosity. Here the shear relaxation modulus has no memory, as represented by a delta function

$$G(t) = \eta\delta(t) \qquad \text{[S2b]}$$

and hence shear relaxation is instantaneous. In contrast, the shear relaxation modulus of an elastic solid is a constant (denoted as G_0), meaning that the stress never relaxes. The result is the expected strain-stress relation

$$\begin{aligned}\tilde{\tau}(t) &= G_0 \int_{-\infty}^{t} dt' \frac{\partial}{\partial t'}\tilde{\varepsilon}(t') \\ &= G_0\tilde{\varepsilon}(t) \qquad \text{[S3]}\end{aligned}$$

Let us further illustrate with a unit-step strain introduced at time t = 0 (Supplementary Fig. 1a):

$$\tilde{\varepsilon}(t) = \Theta(t) \qquad \text{[S4]}$$

Noting the derivative of the Heaviside step function $\Theta(t)$ is a delta function, we find

$$\dot{\tilde{\varepsilon}}(t) = \delta(t) \qquad \text{[S5]}$$

Substituting into Eq [S1], we have

$$\tilde{\tau}(t) = G(t) \qquad \text{[S6]}$$

The shear relaxation modulus thus represents the stress in response to a unit step strain introduced at time t = 0. In the viscous liquid, the stress disappears after t = 0 (Supplementary Fig. 1b, top); i.e., shear relaxation is instantaneously as stated already. In the elastic solid, the

stress, once generated at $t = 0$, stays forever (Supplementary Fig. 1c, top). Viscoelastic fluids fall in between, with shear relaxation occurring over a time period that is between 0 and infinity. An example is a Maxwell fluid, with $G(t)$ given by an exponential function of time (Supplementary Fig. 1d, top). When a Newtonian component and a Maxwell component are added, one arrives at the Jeffreys model of linear viscoelasticity. The combination of two Maxwell components (with different time constants; see Eq. [1] in the main text) makes up the Burgers model.

Another type of shear strain of common interest is a sinusoidal function of time,

$$\tilde{\varepsilon}(t) = \tilde{\varepsilon}_0 e^{i\omega t} \quad \text{[S7a]}$$

The corresponding shear rate is

$$\dot{\tilde{\varepsilon}}(t) = i\omega\tilde{\varepsilon}_0 e^{i\omega t} \quad \text{[S7b]}$$

and the stress is

$$\tilde{\tau}(t) = i\omega\tilde{\varepsilon}_0 e^{i\omega t} \int_{-\infty}^{+\infty} dt' G(t') e^{-i\omega t'} \equiv G^*(\omega)\tilde{\varepsilon}(t) \quad \text{[S8]}$$

The last identity generalizes Eq [S3] and formally defines the complex shear modulus, $G^*(\omega)$. The latter is essentially the Fourier transform of the shear relaxation modulus,

$$G^*(\omega) = i\omega \int_{-\infty}^{+\infty} dt' G(t') e^{-i\omega t'} \quad \text{[S9]}$$

For a viscous liquid (Eq [S2b]), we have

$$G^*(\omega) = i\omega\eta \quad \text{[S10]}$$

which has only an imaginary part (Supplementary Fig. 1b, bottom). On the other hand, for an elastic solid, by comparing Eqs [S3] and [S8], we find

$$G^*(\omega) = G_0 \quad \text{[S11]}$$

which has only a real part (Supplementary Fig. 1c, bottom). In general, viscoelastic fluids have both real and imaginary parts,

$$G^*(\omega) = G'(\omega) + iG"(\omega) \quad \text{[S12]}$$

The real part is called the elastic (or storage) modulus, whereas the imaginary part is called the viscous (or loss) modulus. The elastic and viscous moduli of a Maxwell fluid are shown in Supplementary Fig. 1d, bottom.

Comparison of condensate viscosities by OT and by FRAP

In a previous study [1], we fit fluorescence recovery after photobleaching (FRAP) data to an exponential function

$$F(t) = F(\infty)[1 - e^{-t/\tau_{\mathrm{FR}}}] \quad \text{[S13]}$$

Here we use the resulting time constant (τ_{FR}) to deduce the viscosity inside condensates. According to Soumpasis [2], the half-time, $\tau_{1/2} = (\ln 2)\tau_{\mathrm{FR}}$, and the radius, r_{B}, of the bleached region, can be used to find the diffusion constant of the fluorescently labeled species as

$$D = \frac{0.224 r_{\mathrm{B}}^2}{\tau_{1/2}} = \frac{0.224 r_{\mathrm{B}}^2}{(\ln 2)\tau_{\mathrm{FR}}} \quad \text{[S14]}$$

In the FRAP experiments, r_{B} was kept at 1.39 μm; τ_{FR} was found to be 2.1 ± 0.2, 10.6 ± 0.4, 26.8 ± 1.6, and 105.1 ± 2.3 s, respectively for pK:H, P:H, S:P, and S:L condensates. The fluorescently labeled species was H in the first two cases and S in the last cases. The samples were otherwise the same as in the present study, with the following exceptions. The pK and H concentrations in the pK:H FRAP samples were 50 μM instead of the 100 μM of the present work; the L concentration in the S:L FRAP samples were 2000 μM instead of the 300 μM of the present work. The much higher L concentration makes the condensates denser and hence more viscous. Thus the FRAP-derived viscosity for the S:L condensates should be somewhat higher than the corresponding OT-derived value.

To find the viscosity in condensates, we compare D calculated from τ_{FR} using Eq [S14] to the diffusion constant, D_0, obtained for H or S determined in water. The viscosity of the condensates is

$$\eta = \frac{D_0 \eta_{\mathrm{w}}}{D} = \frac{(\ln 2) D_0 \eta_{\mathrm{w}} \tau_{\mathrm{FR}}}{0.224 r_{\mathrm{B}}^2} \quad \text{[S15]}$$

where η_{w} = 8.9 × 10^{-4} Pa s is the viscosity of water at 25 °C. For H, D_0 for a polymer fraction with molecular weight around 18 kD (as in our samples) was approximately 60 μm^2/s at 20 °C [3]. Using the Stokes-Einstein relation and the viscosities of water, D_0 for H at 25 °C can be found to be 69 μm^2/s. For S in water, we use a scaling relation between D_0 (in μm^2/s at 20 °C) and molecular weight (M, in Dalton), $10^4/D_0 = 4.0M^{1/3} - 6.8$, derived for globular proteins [4], along with M = 42849 Dalton to find D_0 = 75 μm^2/s. The latter translates into D_0 = 87 μm^2/s at 25 °C.

Shear thickening and thinning in droplet shape recovery

In a recent theoretical study [5], the effects of viscoelasticity were investigated by solving the fluid-dynamics equations governing the shape recovery of droplets upon deformation. The theoretical results for shape recovery dynamics showed that, with a Jeffreys model for linear viscoelasticity (Eq [1] in the main text, with $\tau_0 \to 0$), droplets exhibit shear thickening at short τ_1 (shear relaxation time) but shear thinning at long τ_1. Qualitatively, this is the characteristic behavior of associative polymers and also exactly what we observed on the four types of macromolecular droplets, with S:P and S:L as short-τ_1 examples whereas pK:H and P:H as long-τ_1 examples (Fig. 4d). The qualitative agreement of the theoretical calculations and experimental observations lends strong support to our conclusion that shear relaxation provides the governing measure of condensate dynamics, with the shear relaxation time (τ_1) as a key determinant.

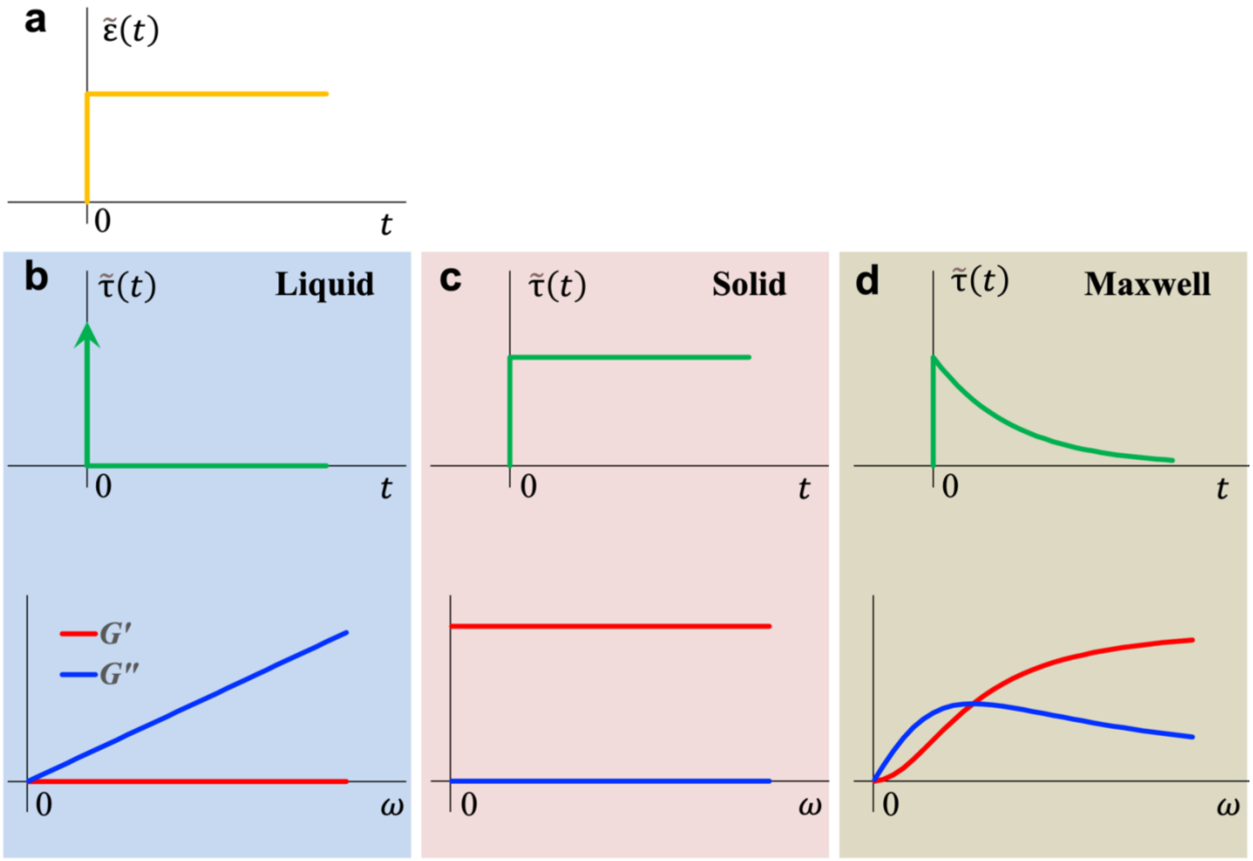

Supplementary Fig. 1 Shear relaxation of liquids, solids, and viscoelastic fluids. **a** A unit-step shear strain introduced at time t = 0. **b-d** Top: the resulting stress inside a viscous liquid, an elastic solid, or a Maxwell fluid. Bottom: the corresponding elastic and viscous moduli. All axes have a linear scale.

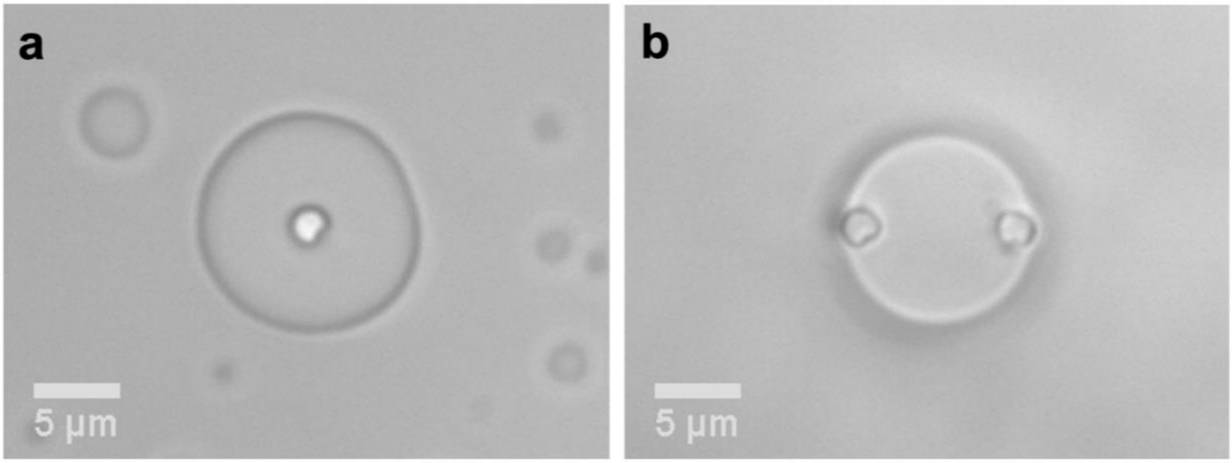

Supplementary Fig. 2 Images of trapped beads at the center or poles of a droplet before measurements. **a** Set up for measuring viscoelasticity. This experiment was repeated on 3 to 4 different droplets; for each droplet, measurements were carried at 9 to 10 oscillation frequencies. **b** Set up for measuring interfacial tension. This experiment was repeated on 10 to 13 different droplets.

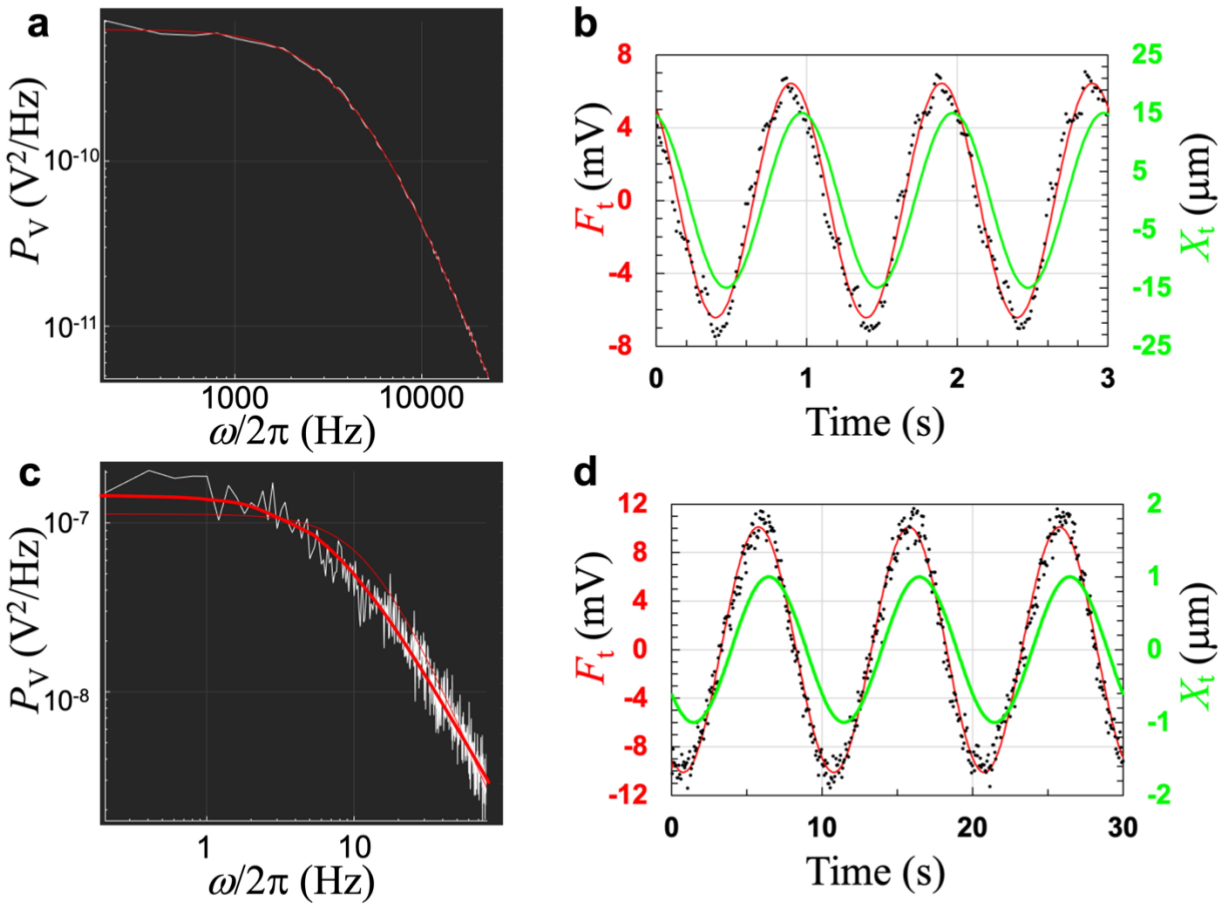

Supplementary Fig. 3 Active-passive calibration, demonstrating that the trap stiffness is unchanged inside macromolecular droplets. **a** Power spectrum of a bead trapped in deionized water. The white trace displays the data; the red trace is a fit to the Lorentzian function (Eq [10] in Methods), with $\omega_c/2\pi$ = 3273 Hz. **b** Time traces of the trap position (X_t; green) and trapping force (F_t; black) on the same bead in water. The trapping force was smoothed by moving average over a 12.8-ms window. A fit of the force trace to a cosine function of time is shown in red. The oscillation frequency ($\omega/2\pi$) was 1 Hz; the amplitude of the trap position was 15 μm. The data in (**a**) and (**b**) together yielded a trap stiffness of 319 pN/μm for this particular bead. **c** Power spectrum of a bead trapped inside a pK:H droplet. The white trace displays the data; the red trace is a fit to a stretched Lorentzian function (Eq [13] in Methods), with α = 1.55 and $\omega_{\text{eff}}/2\pi$ = 6.5 Hz. **d** Time traces of the trap position (X_t; green) and trapping force (F_t; black) on the same bead inside the pK:H droplet. The trapping force was smoothed by moving average over a 64-ms window. A fit of the force trace to a cosine function of time is shown in red. The oscillation frequency was 0.1 Hz; the amplitude of the trap position was 1 μm. The data in (**c**) and (**d**) together yielded a trap stiffness of 300 pN/μm.

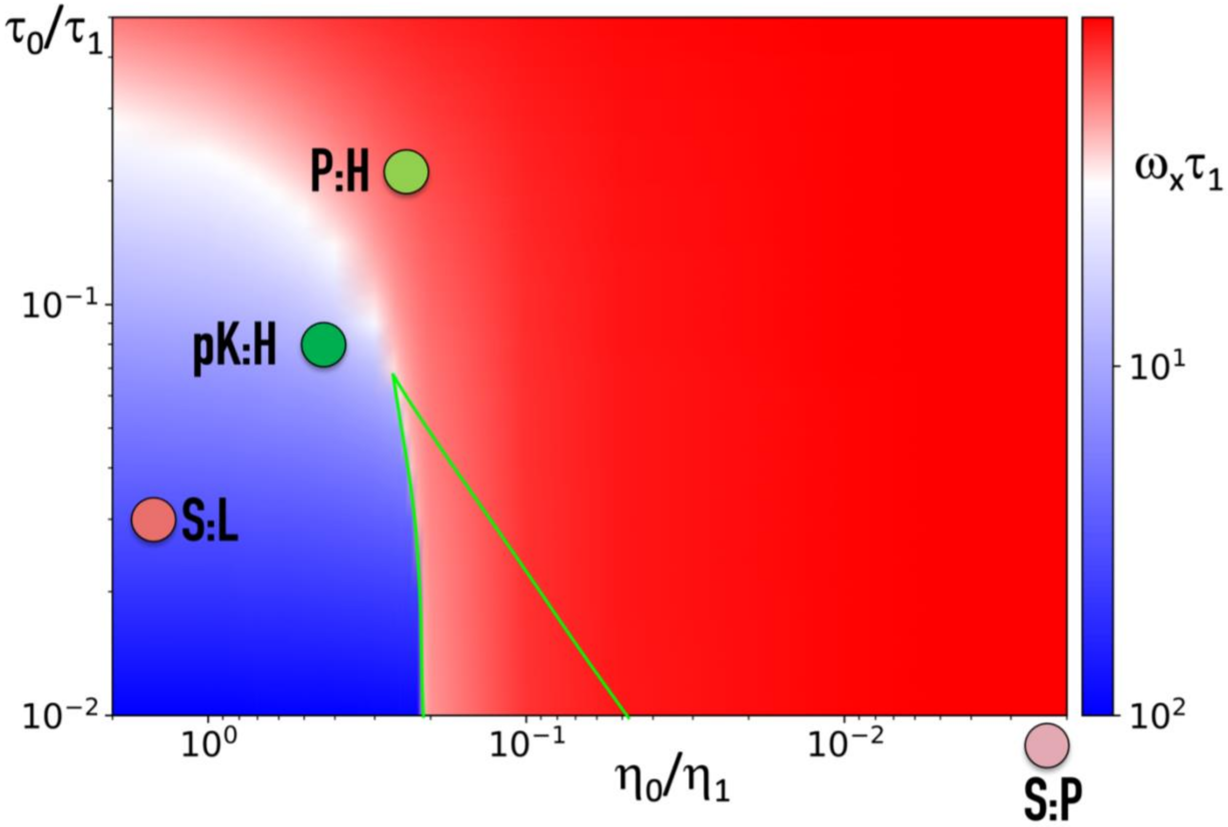

Supplementary Fig. 4 Dependence of the crossover frequency ω_x on η_0/η_1 and τ_0/τ_1. The value of $\omega_x\tau_1$ is displayed by color, according to the scale on the right. The pK:H, P:H, S:P, and S:L condensates are located on the map according to the measured η_0/η_1 and τ_0/τ_1; S:P has $\tau_0/\tau_1 = 0$ and is placed along the abscissa. Red regions have ω_x close to $1/\tau_1$ whereas blue regions have ω_x close to $1/\tau_0$. Inside the triangular region bordered by green curves, $G'(\omega)$ and $G"(\omega)$ cross each other three times; only the smallest crossover frequency is displayed.

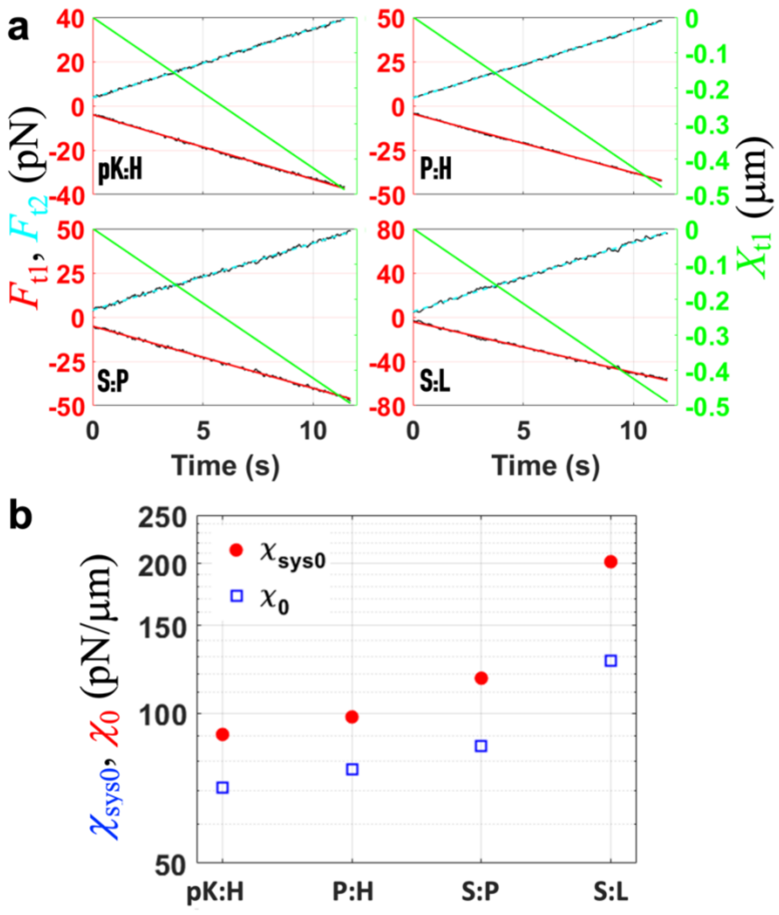

Supplementary Fig. 5 Raw data for interfacial tension measurements by stretching droplets. **a** Typical traces of the trap 1 position (X_{t1}; green) and traps 1 and 2 forces (F_{t1} and F_{t2}; black), one set each from pK:H, P:H, S:P, and S:L droplets. The plot for pK:H is the same as displayed in Fig. 2d. The force traces were smoothed by moving average over a 64-ms window; linear fits are overlaid. The slopes of the linear fits are used in Eq [39] of Methods to obtain χ_{sys0}; this χ_{sys0} along with the stiffnesses of the two traps (450 to 750 pN/μm) are used in Eq [38] to obtain χ_0. **b** Plot of χ_{sys0} and χ_0 from (**a**) for the four droplets, showing successive increases from pK:H to P:H to S:P to S:L.

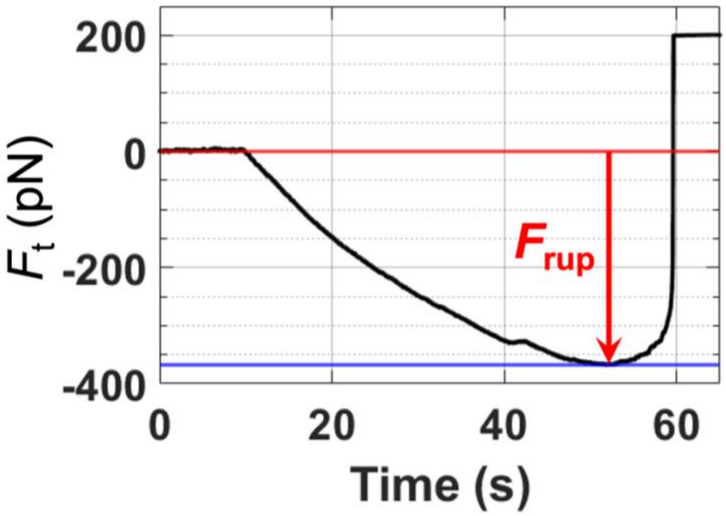

Supplementary Fig. 6 Rupture force of a pK:H droplet. The trap 1 force was smoothed by moving average over a 64-ms window. For this particular droplet, the rupture force was 367.9 pN, corresponding to an interfacial tension of 53.2 pN/μm according to Eq [43] in Methods.

Supplementary References

1. Ghosh, A., Zhou, H. X. Determinants for Fusion Speed of Biomolecular Droplets. *Angew Chem Int Ed* **59**, 20837-20840 (2020).

2. Soumpasis, D. M. Theoretical analysis of fluorescence photobleaching recovery experiments. *Biophys J* **41**, 95-97 (1983).

3. Pavlov, G., Finet, S., Tatarenko, K., Korneeva, E., Ebel, C. Conformation of heparin studied with macromolecular hydrodynamic methods and X-ray scattering. *Eur Biophys J* **32**, 437-449 (2003).

4. Zhou, H. X. Calculation of translational friction and intrinsic viscosity. II. Application to globular proteins. *Biophys J* **69**, 2298-2303 (1995).

5. Zhou, H.-X. Shape Recovery of Deformed Biomolecular Droplets: Dependence on Condensate Viscoelasticity. *J Chem Phys*, in press (2021).
