# Supplementary material for: Shear relaxation governs fusion dynamics of biomolecular condensates
Source: Nat Commun. 2021 Oct 13;12:5995. doi: 10.1038/s41467-021-26274-z (PMC8514506; doi:10.1038/s41467-021-26274-z)
Supplement: Supplementary file 2 — Description of Additional Supplementary Files [file 41467_2021_26274_MOESM2_ESM.pdf]

Description of Additional Supplementary Files

File Name: Supplementary Movie 1
Description: **Oscillating a trapped bead inside a settled pK:H droplet.** The trapped bead is seen as a white spot inside the droplet, oscillating at a frequency of 1 Hz.

File Name: Supplementary Movie 2
Description: **Stretching of a pK:H droplet suspended by two trapped beads at the opposite poles.** The trapped bead on the right is fixed in place while the trapped bead on the left is pulled toward the left, with a total displacement of 0.5 µm. The pulling speed is slightly less than 0.05 µm/s. The movie is played at four times the recording speed.

File Name: Supplementary Movie 3
Description: **Surface rupture of a pK:H droplet by pulling a trapped bead from inside.** The movie is played at eight times the recording speed.
