# Supplementary material for: Shear relaxation governs fusion dynamics of biomolecular condensates
Source: Nat Commun. 2021 Oct 13;12:5995. doi: 10.1038/s41467-021-26274-z (PMC8514506; doi:10.1038/s41467-021-26274-z)
Supplement: Supplementary file 6 — Reporting Summary [file 41467_2021_26274_MOESM6_ESM.pdf]

nature research

Corresponding author(s): Huan-Xiang Zhou

Last updated by author(s): Sep 23, 2021

Reporting Summary

Nature Research wishes to improve the reproducibility of the work that we publish. This form provides structure for consistency and transparency in reporting. For further information on Nature Research policies, see our Editorial Policies and the Editorial Policy Checklist.

Statistics

For all statistical analyses, confirm that the following items are present in the figure legend, table legend, main text, or Methods section.

n/a	Confirmed	
☐	☒	The exact sample size (n) for each experimental group/condition, given as a discrete number and unit of measurement
☐	☒	A statement on whether measurements were taken from distinct samples or whether the same sample was measured repeatedly
☒	☐	The statistical test(s) used AND whether they are one- or two-sided<br>*Only common tests should be described solely by name; describe more complex techniques in the Methods section.*
☒	☐	A description of all covariates tested
☒	☐	A description of any assumptions or corrections, such as tests of normality and adjustment for multiple comparisons
☐	☒	A full description of the statistical parameters including central tendency (e.g. means) or other basic estimates (e.g. regression coefficient) AND variation (e.g. standard deviation) or associated estimates of uncertainty (e.g. confidence intervals)
☒	☐	For null hypothesis testing, the test statistic (e.g. F, t, r) with confidence intervals, effect sizes, degrees of freedom and P value noted<br>*Give P values as exact values whenever suitable.*
☒	☐	For Bayesian analysis, information on the choice of priors and Markov chain Monte Carlo settings
☒	☐	For hierarchical and complex designs, identification of the appropriate level for tests and full reporting of outcomes
☒	☐	Estimates of effect sizes (e.g. Cohen's d, Pearson's r), indicating how they were calculated

Our web collection on statistics for biologists contains articles on many of the points above.

Software and code

Policy information about availability of computer code

Data collection	Data collection was made on a commercial instrument, manufactured by LUMICKS.
Data analysis	Custom codes in MATLAB (Version R2018b) were written to analyze experimental data. Additional curve fitting was done in GNUPlot (Version 5.0 patchlevel 3). Codes are posted on GitHub at https://github.com/hzhou43/viscoelasticity-of-biomolecular-condensates/Codes.

For manuscripts utilizing custom algorithms or software that are central to the research but not yet described in published literature, software must be made available to editors and reviewers. We strongly encourage code deposition in a community repository (e.g. GitHub). See the Nature Research guidelines for submitting code & software for further information.

Data

Policy information about availability of data

All manuscripts must include a data availability statement. This statement should provide the following information, where applicable:

- Accession codes, unique identifiers, or web links for publicly available datasets
- A list of figures that have associated raw data
- A description of any restrictions on data availability

Figures 3a and 4b has associated raw data. These data are posted on GitHub at https://github.com/hzhou43/viscoelasticity-of-biomolecular-condensates/Data. Other data in Figure 4a-c are listed in Table 1.

Field-specific reporting

Please select the one below that is the best fit for your research. If you are not sure, read the appropriate sections before making your selection.

- [x] Life sciences
- [ ] Behavioural & social sciences
- [ ] Ecological, evolutionary & environmental sciences

For a reference copy of the document with all sections, see nature.com/documents/nr-reporting-summary-flat.pdf

Life sciences study design

All studies must disclose on these points even when the disclosure is negative.

Sample size	Our sample consisted of replicate measurements of a given property, instead of a group of study subjects.<br>We did not perform a calculation on sample size, i.e., total number of replicate measurements. The number of replicate measurements was chosen based on the variability of the acquired data. The replicate number for each property was at least three, and as high as 20, and in each case was judged adequate, based on the observation that the standard error of the mean was typically less than 10% of the mean value.
Data exclusions	None
Replication	As explained above, the number of replicate measurements ranged from 3 to 20. All attempts at replication were successful.
Randomization	Again, our sample consisted of replicate measurements of a given property. All the replicate measurements were independent -- in that sense our sample was inherently randomized.
Blinding	Because our sample consisted of replicate measurements, the concept of blinding does not apply.

Reporting for specific materials, systems and methods

We require information from authors about some types of materials, experimental systems and methods used in many studies. Here, indicate whether each material, system or method listed is relevant to your study. If you are not sure if a list item applies to your research, read the appropriate section before selecting a response.

Materials & experimental systems

n/a	Involved in the study	
x		Antibodies
x		Eukaryotic cell lines
x		Palaeontology and archaeology
x		Animals and other organisms
x		Human research participants
x		Clinical data
x		Dual use research of concern

Methods

n/a	Involved in the study	
x		ChIP-seq
x		Flow cytometry
x		MRI-based neuroimaging
